# KIAA1199 (CEMIP) regulates adipogenesis and whole-body energy metabolism

**DOI:** 10.1038/s41413-025-00415-2

**Published:** 2025-04-02

**Authors:** Li Chen, Kaikai Shi, Nicholas Ditzel, Weimin Qiu, Michaela Tencerova, Louise Himmelstrup Dreyer Nielsen, Florence Figeac, Alexander Rauch, Yuhang Liu, Jiuyuan Tao, Veronika Sramkova, Lenka Rossmeislova, Greet Kerckhofs, Tatjana N. Parac-Vogt, Sébastien de Bournonville, Thomas Levin Andersen, Mikael Rydén, Moustapha Kassem

**Affiliations:** 1 Guangxi Key Laboratory of Tumor Immunology and Microenvironment Regulation, Guilin Medical University, Guilin, Guangxi 541199 China; 2 Department of Endocrinology and Metabolism, Endocrine Research Laboratory (KMEB), Odense University Hospital & University of Southern Denmark, Odense, Dk-5230 Denmark; 3 Department of Pathophysiology, Centre for Research on Diabetes, Metabolism and Nutrition, Third Faculty of Medicine, Charles University, Prague, Czech Republic; 4 Biomechanics Lab, Institute of Mechanics, Materials, and Civil Engineering, KU Leuven, Leuven, Belgium; 5 Biomechanics Section, Department of Mechanical Engineering, KU Leuven, Leuven, Belgium; 6 Institute of Pathology, University of Southern Denmark, Odense, Denmark; 7 Department of Medicine (H7), Karolinska Institute, Karolinska University Hospital, Stockholm, Sweden; 8 Khalifa University, College of Medicine and Health Sciences & Biotechnology Center (BTC), Abu Dhabi, United Arab Emirates

**Keywords:** Endocrine system and metabolic diseases, Fat metabolism

## Abstract

An increasing number of studies have characterized the bone as an endocrine organ, and that bone secreted factors may not only regulate local bone remodeling, but also other tissues and whole-body metabolic functions. The precise nature of these regulatory factors and their roles at bridging the bone, bone marrow adipose tissue, extramedullary body fat and whole-body energy homeostasis are being explored. In this study, we report that KIAA1199, a secreted factor produced from bone and bone marrow, previously described as an inhibitor of bone formation, also plays a role at promoting adipogenesis. KIAA1199-deficient mice exhibit reduced bone marrow adipose tissue, subcutaneous and visceral fat tissue mass, blood cholesterol, triglycerides, free fatty acids and glycerol, as well as improved insulin sensitivity in skeletal muscle, liver and fat. Moreover, these mice are protected from the detrimental effects of high-fat diet feeding, with decreased obesity, lower blood glucose and glucose tolerance, as well as decreased adipose tissue inflammation, insulin resistance and hepatic steatosis. In human studies, plasma levels of KIAA1199 or its expression levels in adipose tissue are positively correlated with insulin resistance and blood levels of cholesterol, triglycerides, free fatty acids, glycerol, fasting glucose and HOMA-IR. Mechanistically, KIAA1199 mediates its effects on adipogenesis through modulating osteopontin-integrin and AKT / ERK signaling. These findings provide evidence for the role of bone secreted factors on coupling bone, fat and whole-body energy homeostasis.

## Introduction

It is increasingly recognized that the skeleton exerts additional biological functions beyond its canonical role of providing biomechanical support and regulation of mineral homeostasis. For instance, it acts as an endocrine organ that secretes endocrine factors in response to changes of whole-body energy metabolism,^1^ e.g. osteocalcin,^2^ lipocalin-2^3^ and delta-like 1 (Dlk-1)^4^ that provide a link between bone remodeling activities and whole body energy requirements. A common biological feature of these factors is that they regulate energy metabolism through interaction with insulin signaling (osteocalcin, Dlk1) or energy intake (lipocalin-2). From an evolutionary perspective, the substantial energy needed for maintenance of life-long bone remodeling, requires the coordinated functions of bone remodeling and whole-body energy metabolism.^2,5^

Bone marrow stromal (skeletal, or mesenchymal) stem cells (BMSCs) play a central role in bone remodeling due to their ability to differentiate into bone forming osteoblasts and energy-storing bone marrow adipose tissue (BMAT).^6^ We have previously employed quantitative proteome analysis in order to identify the secreted proteins, ‘secretome’ of human BMSC (hBMSC), and identified a complex profile of secreted proteins with annotated functions that extend beyond regulation of bone remodeling, and with possible variety of biological effects e.g. tissue growth, immune regulation, and regulation of energy metabolism.^7–9^

Among the secreted proteins, we identified KIAA1199, a 150 kD protein encoded by the CEMIP gene (cell migration inducing hyaluronan binding protein), located on human chromosome 15q25.1.^10^ At the molecular level, KIAA1199 binds to and degrades hyaluronic acid (HA) and plays a role in extracellular matrix remodeling.^11,12^ KIAA1199 is present in peripheral blood and its levels have been employed as a prognostic biomarker for inflammatory arthritis^13^ and in certain types of cancer^14^ corroborating the hypothesis of possible systemic functions.

We have previously reported that KIAA1199 is highly expressed and secreted by bone and marrow stromal stem cells, it enhances hBMSC motility and migration in vitro,^15^ as well as regulates osteoblast functions, bone formation and bone regeneration in vivo.^16^ In the present study, we investigated the biological role of KIAA1199 in adipocyte differentiation and its impact on fat formation and whole-body energy metabolism in vivo by employing KIAA1199 knockout (KO) mice under both normal and high-fat diet feeding. In addition, we measured KIAA1199 blood levels in multiple cohorts of patients with obesity and metabolic dysfunction. Our results demonstrate that the role of KIAA1199 regulating bone formation is associated with regulation of bone marrow and extramedullary fat tissue formation and whole-body energy metabolism.

## Results

### KIAA1199 is a circulating factor highly expressed in bone marrow stromal stem cells, and significantly regulates adipocyte (AD) differentiation of hBMSC

We have previously identified KIAA1199 as a protein secreted by hBMSC,^7^ and its expression is mostly enriched in bone and bone marrow in mice.^16^ Here, we confirmed in human tissues that KIAA1199 expresses in brain, pancreas and testis, and significantly higher levels in bone and cultured bone marrow stromal stem cells (hBMSC) (Fig. S1a). We also observed similar results in mouse tissues (Fig. S1b). In situ hybridization of human bone biopsies showed that KIAA1199 is not only highly expressed in osteoblast and osteoprogenitor cells at the bone remodeling sites as we have previously reported,^15,16^ but also expressed in hBMSC adjacent to mature bone marrow adipocytes (Fig. S1c). RNA-seq, real time PCR and Western blot analysis determined that KIAA1199 is progressively downregulated during AD differentiation of hBMSC (Fig. S1d–h). Monitoring KIAA1199 expression at single cell resolution on Day 0 and Day 7 during AD differentiation of hBMSC reported in our previous study,^17^ we observed a decreased expression and secretion of KIAA1199 during adipogenesis progression (Fig. S1i, j). Moreover, using our previous genome-wide maps of enhancer activity data^17^ and chromatin interaction data,^18^ we found a progressive decreased H3K27-acetylation, mediator occupancy and connectivity with the downstream super-enhancer region to the KIAA1199 promoter region during AD differentiation (Fig. S1k), suggesting a inhibition of KIAA1199 during AD process.

**Fig. 1:**
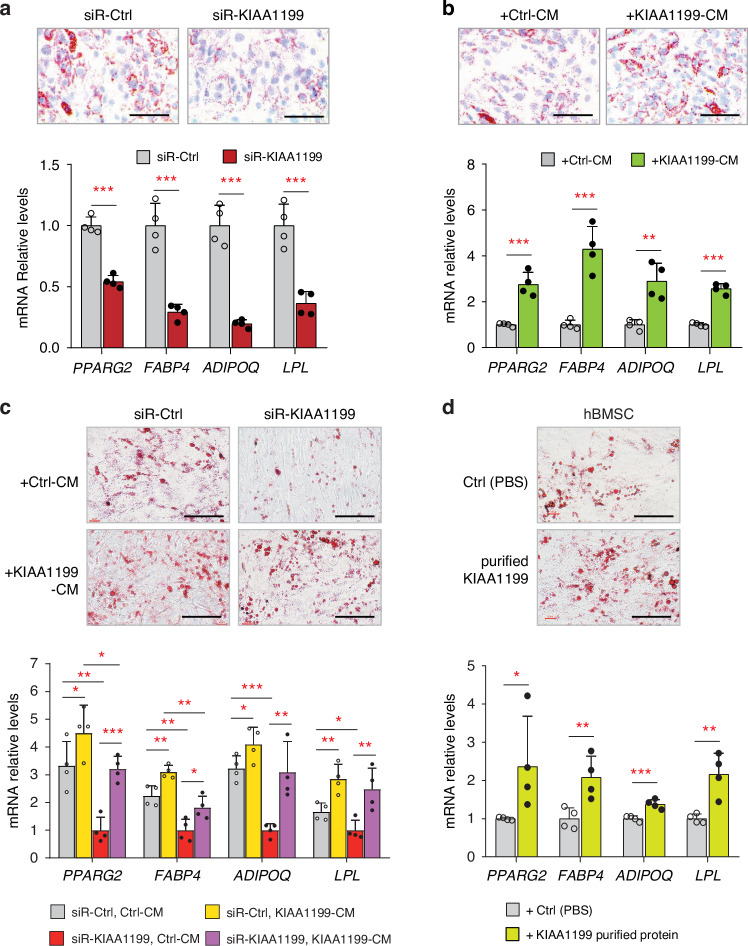
KIAA1199 regulates adipocyte (AD) differentiation of human bone marrow stromal stem cells (hBMSC). **a** siRNA-mediated knock down of KIAA1199 inhibited AD differentiation. hBMSCs transfected with specific KIAA1199 siRNAs (siR-KIAA1199) or with non-target control siRNA (siR-Ctrl) and induced to AD differentiation; **b** Conditioned media (CM) from cells overexpressing KIAA1199 (KIAA1199-CM) or control vehicle cells (Ctrl-CM) were added into hBMSC during AD differentiation; **c** KIAA1199-CM or Ctrl-CM were added into hBMSC that transfected with siR-Ctrl or siR-KIAA1199 during AD differentiation; **d** KIAA1199 protein purified from CM was added into hBMSC during AD differentiation. Oil red O staining was performed at day 14 (**a, c**) or day 10 (**b, d**) for lipid-filled adipocytes. The expressions of AD marker genes (*PPARg2, FABP4, ADIPOQ and LPL*) were analyzed by qPCR. Scale bar = 100 µm. At least three independent experiments had run for tests. All data are expressed as means ± SD. Statistical difference is determined by one-way ANOVA with Dunnett’s multiple tests to compare with control group or two-tailed unpaired Student’s *t*-test between two groups, *P < 0.05, **P < 0.01 and ***P < 0.001, *n* ≥ 3

To examine the regulatory effects of KIAA1199 on AD differentiation, we firstly inhibited the expression of KIAA1199 by specific siRNAs in hBMSC, that reduced the KIAA1199 expression by more than 85% during 14 days of in vitro AD differentiation (Fig. S2a, b), and led to significantly impaired AD differentiation, evidenced by fewer lipid-filled, Oil red O positive mature adipocytes and decreased expression levels of adipocyte specific genes: *PPARG2, FABP4, ADIPOQ* and *LPL* (Fig. 1a). Conversely, when treating hBMSC with soluble KIAA1199 present in the conditioned medium (CM) from KIAA1199 overexpressing cells^15^ (Fig. S2c), AD differentiation was enhanced (Fig. 1b). Adding CM from KIAA1199 overexpression cells partly rescued the observed inhibition of AD differentiation in KIAA1199-deficient hBMSC (Fig. 1c). Moreover, adding KIAA1199 purified protein from mammalian cells (Fig. S2d) enhanced the AD differentiation of hBMSC, similar to what observed when treating cells with CM of KIAA1199-overexpression cells (Fig. 1d). These results demonstrate that KIAA1199 acts as a positive regulator of bone marrow adipocyte differentiation.

### KIAA1199 KO mice exhibit reduced bone marrow adipose tissue (BMAT) and extramedullary fat mass by inhibiting adipocyte differentiation

**Fig. 2:**
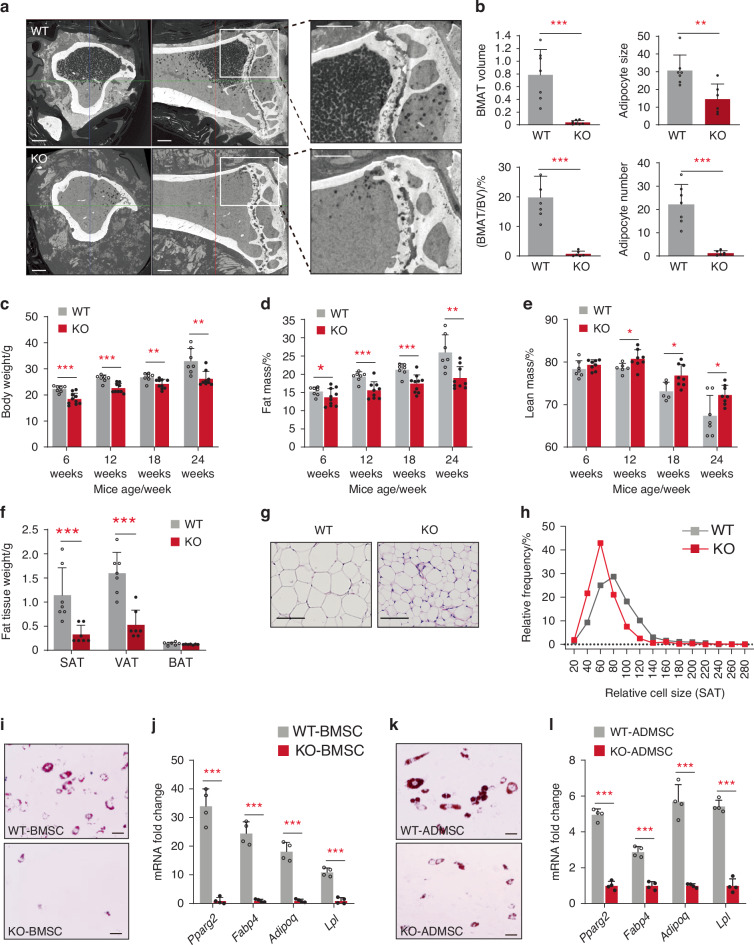
KIAA1199 deficient mice (KO) display reduced marrow and extramedullary fat mass and impaired adipocyte differentiation. **a** Representative Hf-POM-based CE-µCT scan showing marrow adipocytes as black spots, scale bar = 5 μm. **b** Quantifying 3D bone marrow adipose tissue (BMAT) volume (mm^3^), BMAT volume/bone marrow volume (%, V/V), BMAT adipocyte number (×10^3^/mm^3^) and size (µm^3^ × 10^3^/mm^3^), *n* = 7/group. **c**–**e** Body weight(g), fat mass(%), lean mass(%) of KO and WT female mice measured by DEXA scan, *n* = 7–10. **f** Weight of fat mass depots in female KO mice and WT control: SAT subcutaneous adipose tissue, VAT visceral adipose tissue, BAT brown adipose tissue, *n* = 8. Representative histological H&E-stained of subcutaneous adipose tissue (SAT) from female KO and WT mice (**g**), SAT adipocyte sizes were measured by ImageJ (Adiposoft), the size frequencies were analyzed by GraphPad (**h**), *n* = 6/group. Mouse bone marrow stromal stem cells (mBMSC) (**i, j**) and mouse stromal cells derived from SAT (mADMSC) (**k, l**) in female KO and WT mice (*n* = 5) subjected to in vitro AD differentiation. Formation of lipid-filled adipocytes stained with Oil red O (**i, k**) and expression of AD marker genes (*Pparg2, Fabp4, Adipoq* and *Lpl*) determined by qPCR, *n* = 4 (**j, l**). Scale bar (**g, i, k**) = 100 µm. Data are expressed as means ± SD, the comparison between WT and KO groups are performed by two-tailed unpaired Student’s *t*-test, *P < 0.05, **P < 0.01, ***P < 0.001

To further investigate the in vivo effects of KIAA1199 on adipose tissue formation, we evaluated the changes in fat depots size in KIAA1199 deficient (KIAA1199 KO) mice created by CRISPR-Cas9 technology.^16^ BMAT in the proximal metaphysis of the tibia were measured using polyoxometalate-based contrast-enhanced microCT (Hf-POM-based CE-µCT).^19^ Compared to age and gender matched wild-type controls under normal diet, KIAA1199 KO female mice exhibited significantly reduced BMAT volume, BMAT cell number and adipose cell size (Fig. 2a, b). Also, KIAA1199 KO female mice have reduced body weight (Fig. 2c and Fig. S3a) and whole-body fat mass (Fig. 2d and Fig. S3b), but increased lean body mass fraction (Fig. 2e and Fig. S3c). Specifically, subcutaneous adipose tissue (SAT) and visceral adipose tissue (VAT) were significantly reduced while interscapular brown adipose tissue (BAT) mass did not change (Fig. 2f). Similar phenotype was also observed in male mice (Fig. S3d-f and Fig. S4). Histological analysis revealed that KIAA1199-deficiency resulted in reduced adipocyte size of SAT, VAT and BAT (Fig. 2g, h and Fig. S5).

To determine whether the observed in vivo reduced fat mass in KO mice caused by the impaired adipocyte (AD) differentiation, primary murine bone marrow MSC (mBMSC) and primary murine adipose tissue derived stromal cells (mADMSC) were isolated from KIAA1199 KO and age-matched WT mice and induced to in vitro AD differentiation. Significant impairment of AD differentiation was observed in both cell types in KIAA1199 deficient cells, with decreased number of mature Oil red O positive adipocytes (Fig. 2i, k) and significantly reduced expression of adipocytic gene markers: *Pparg2, Fabp4, adipoq and Lpl* (Fig. 2j, l).

### KIAA1199 regulates glucose and lipid metabolism in mice

**Fig. 3:**
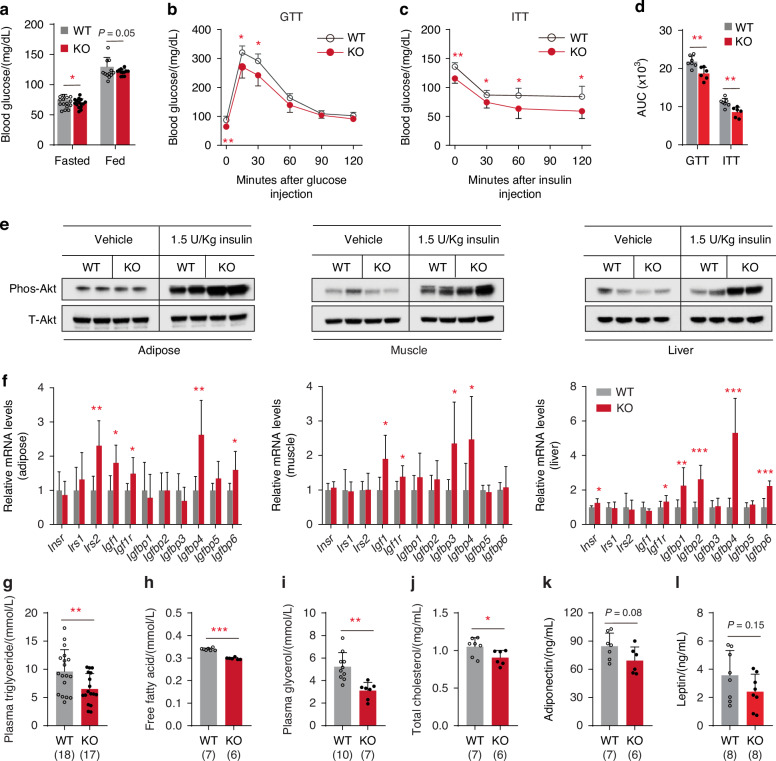
KIAA1199 regulates glucose homeostasis and lipid metabolism. **a** Plasma levels of glucose under fasting and fed in female KIAA1199 KO and WT mice, *n* = 12–15/group. The glucose tolerance test (GTT) (**b**), insulin tolerance test (ITT) (**c**), the calculated area under the curve (AUC) for GTT and ITT (**d**) were checked in female KO (*n* = 6) and WT (*n* = 7) mice. **e** Western blot analysis of phosphorylated Akt (P-Akt, p473) and total Akt (T-Akt) in adipose, muscle and liver tissues after 7 min intraperitoneal injection of insulin or vehicle in female mice, *n* = 3. **f** Steady-state expressions of genes associated with insulin signaling in adipose, muscle and liver from female mice, *n* = 7. **g**–**l** Plasma levels of triglycerides, free fatty acids, glycerol, total cholesterol, adiponectin and leptin measured by ELISA in female mice, *n* ≥ 7. Three independent experiments had run for test, the representative data are shown. All data are represented as mean ± SD, the comparison between WT and KO groups are performed by two-tailed unpaired Student’s *t*-test. *P < 0.05, **P < 0.01, ***P < 0.001

Since KIAA1199 KO mice exhibited significant reduction in fat mass, we examined whether this phenotype is associated with changes in whole-body energy metabolism. KIAA1199 KO mice under normal diet did not exhibit significant alterations in food intake (Figs. S6, S7a and S8a), ambulatory activity, oxygen consumption, carbon dioxide production or respiratory exchange ratio (RER) as determined by studies performed in metabolic cages (Figs. S7 and S8). Under normal diet, KIAA1199 KO mice were not observed significant changes in liver histology (Fig. S9a) or fasting serum insulin levels (Fig. S9b). Moreover, KIAA1199 KO mice have similar insulin secretion in glucose stimulated insulin secretion (GSIS) test (Fig. S9c). Consistently, the number and size of β-cell in pancreatic islets in KIAA1199 KO compared to WT controls are similar (Fig. S9d–g). On the other hand, the fasting and random blood glucose levels were reduced in KIAA1199 KO (Fig. 3a). Glucose tolerance test (GTT) and insulin tolerance test (ITT) revealed that KIAA1199 KO mice exhibited enhanced insulin sensitivity (Fig. 3b–d). Examining the phosphorylation of Akt in peripheral tissues following intraperitoneal injection of insulin, revealed higher activation of Akt in adipose, muscle and liver tissues in KIAA1199 KO mice (Fig. 3e). These data suggest that there is increased insulin sensitivity in insulin target tissues in the absence of KIAA1199. Consistently, expression of the genes associated with insulin signaling e.g., *Igf1, Igf1r* and *Igfbps* were also increased in KIAA1199 KO adipose, muscle and liver tissues (Fig. 3f and Fig. S10) compared to WT. Furthermore, we observed significant reduction of plasma levels of triglycerides, free fatty acids, glycerol and total cholesterol in KIAA1199 KO mice (Fig. 3g–j), while we did not observe significant changes in plasma levels of total adiponectin or leptin (Fig. 3k, l). These data suggest that KIAA1199 exerts significant effects on whole-body glucose and lipid metabolism with modulating target tissues’ insulin sensitivity.

### KIAA1199 KO mice are protected from the deleterious effects of high-fat diet (HFD) feeding

**Fig. 4:**
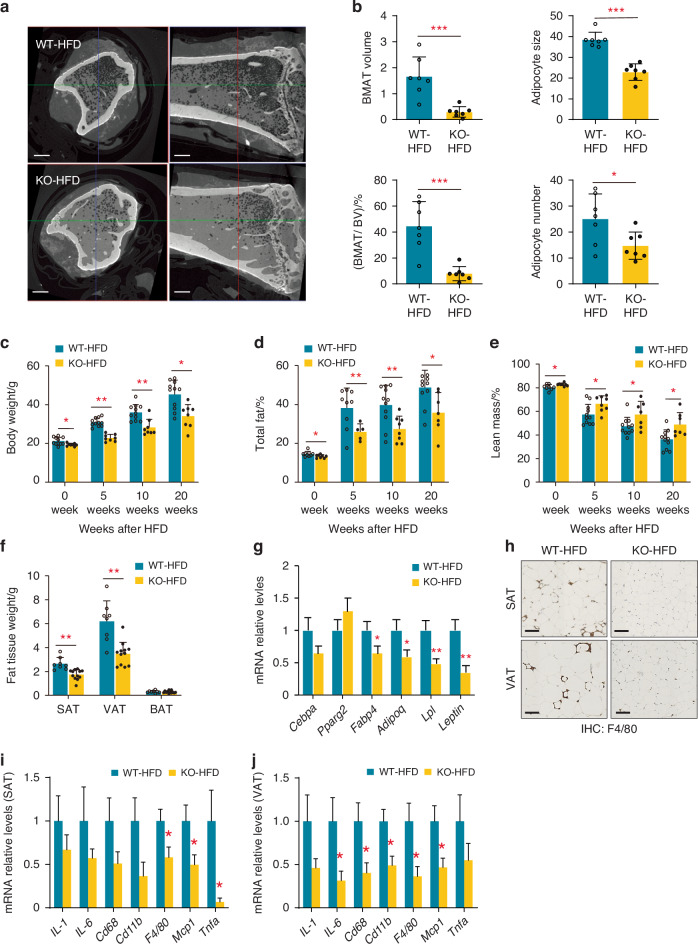
KIAA1199 knockout (KO) mice are protected from obesity following high-fat-diet (HFD) feeding. KIAA1199 KO and WT female mice received 20 weeks high fat diet (HFD). **a** Hf-POM-based CE-µCT scan of the tibiae show bone marrow adipocytes tissue (BMAT) as black spots, scale bar = 5 μm. **b** Quantitation of BMAT volume (mm^3^), BMAT volume ratio of bone marrow (%, V/V), BMAT number (×10^3^/mm^3^) and size (µm^3^ × 10^3^/mm^3^) were analyzed, *n* = 7. **c**–**e** Body weight (g), fat mass (%), lean mass (%) were measured by DEXA scan, *n* = 7–11. **f** Weight of fat mass depots: SAT subcutaneous fat, VAT visceral fat, BAT brown fat issues. **g** Expression of adipocyte gene markers were determined in VAT by qPCR, *n* = 7, scale bar = 100 µm. **h** The representative immunohistochemistry (IHC) photomicrographs of SAT and VAT with F4/80 antibody for inflammatory cells, following 24 weeks HFD. The inflammation-associated genes (*Il-1*, *Il-6*, *Cd68*, *Cd11b*, *F4/80*, *Mcp1* and *Tnfα*) were analyzed by qPCR in SAT (**i**) and VAT (**j**), *n* = 7. Three independent experiments had run for test, the representative data are shown, data are expressed as means ± SD, the comparison between WT and KO groups are performed by two-tailed unpaired Student’s *t*-test. *P < 0.05, **P < 0.01, ***P < 0.001

As KIAA1199 KO mice exhibited increased insulin sensitivity and reduced fat mass under normal diet, we examined the response of KIAA1199 KO mice to an obesogenic high-fat diet (HFD, 60% of calories from fat). Following 20 weeks of HFD, we observed significant expansion of BMAT toward the central part of the long bone’s diaphysis in female WT mice, which was absent in KIAA1199 KO mice (Fig. 4a, b). In addition, KIAA1199 KO mice gained significantly less weight, less total body fat mass, but higher lean mass compared to WT controls (Fig. 4c–e and Fig. S11). Similar to the observations under normal diet, KIAA1199 KO mice under HFD exhibited reduced SAT and VAT, but not BAT (Fig. 4f) and histological analysis revealed smaller adipocytes in SAT, VAT and BAT (Fig. S12), as well as reduced expression levels of AD-specific genes (Fig. 4g and Fig. S13a). We also observed reduced number of inflammatory cells infiltrate in SAT and VAT detected by F4/80 immunostaining (Fig. 4h), as well as significantly reduced expression of inflammatory gene markers (Fig. 4i, j and Fig. S13b, c).

**Fig. 5:**
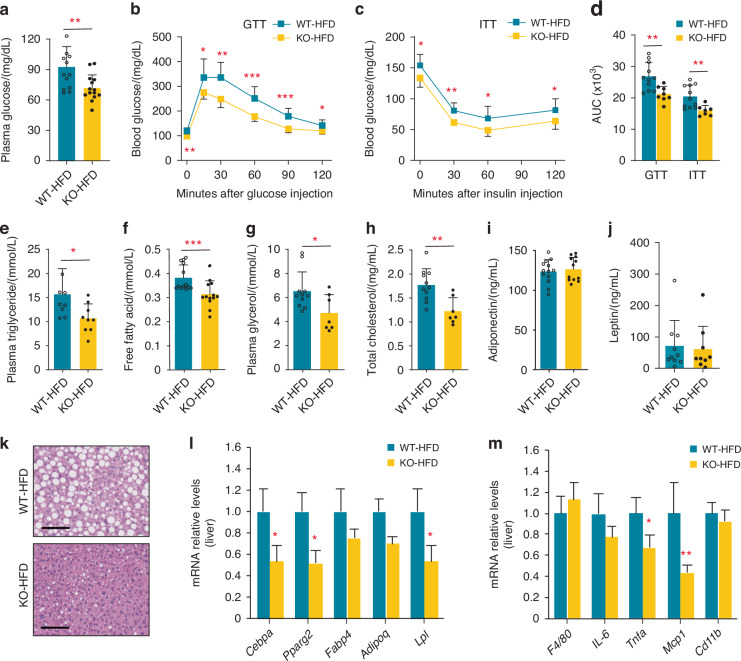
KIAA1199 knockout (KO) mice are protected from the deleterious metabolic effects of high-fat-diet (HFD). KIAA1199 KO and WT female mice (*n* = 7–14/group) following 8–20 weeks high-fat-diet (HFD). Fasting blood glucose (**a**), glucose tolerance test (GTT) (**b**), insulin tolerance test (ITT) (**c**), the area under curve (AUC) for both GTT and ITT tests (**d**) were measured following 10–14 weeks HFD. Plasma triglycerides (**e**), free fatty acids (**f**), glycerol (**g**), total cholesterol (**h**), adiponectin (**i**) and leptin (**j**) levels were measured by ELISA following 20 weeks HFD. **k** Photomicrograph of representative liver tissue sections stained by H&E following 20 weeks HFD, scale bar = 100 µm. Expression of adipocyte marker genes (**l**) and inflammation-associated marker genes (**m**) in the liver tissues measured by q-PCR. Three independent experiments had run for test, the representative data are shown, data are expressed as means ± SD, the comparison between WT and KO groups are performed by two-tailed unpaired Student’s *t*-test, *P < 0.05, **P < 0.01, ***P < 0.001

In vivo metabolic studies including fasting plasma glucose, GTT and ITT demonstrated improved insulin sensitivity in KIAA1199 KO female mice under HFD (Fig. 5a–d). Interestingly, fasting insulin levels (Fig. S14a), the insulin response in GSIS test (Fig. S14b), and the number and size of β-cell in pancreatic islets (Fig. S14c, d) were similar in KIAA1199 KO female mice and WT controls. Similar findings were also observed in male KIAA1199 KO mice (Figs. S14d–f and S15). Moreover, KIAA1199 KO mice exhibited an improved lipid profile with lowered plasma levels of triglycerides, fatty acids, glycerol and total cholesterol (Fig. 5e–h), while plasma adiponectin and leptin levels did not differ between KIAA1199 KO and WT mice under HFD (Fig. 5i, j). Interestingly, HFD induced hepatic steatosis in WT mice, which was significantly reduced in KIAA1199 KO as evidenced by lower liver weight and liver lipid accumulation (Fig. 5k and Fig. S16a–d), and associated with lower expression levels of both AD gene markers (Fig. 5l and Fig. S16e) and inflammatory gene markers (Fig. 5m and Fig. S16f) in livers.

Female KIAA1199 KO did not differ in the food consumption compared to WT controls (Figs. S17a and S18a), but exhibited increased in ambulatory activity (Fig. S18b, c) and a trend towards increased respiratory exchange ratio (RER) in metabolic cages (Fig. S18i). Similar phenotypes were detected in male mice under HFD (Figs. S17b and S19). Taken together, these data demonstrate that KIAA1199 KO mice are protected from the deleterious metabolic effects of HFD feeding.

### KIAA1199 expression and plasma levels correlate with obesity and metabolic syndrome phenotype in humans

**Fig. 6:**
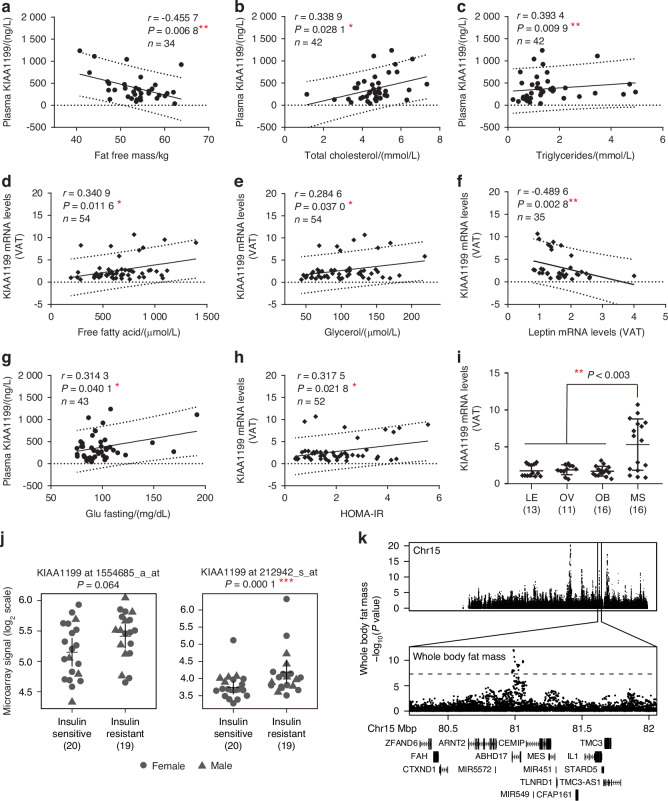
KIAA1199 gene expression and plasma levels correlate with obesity and insulin resistance phenotype in humans. Plasma KIAA1199 was measured in a cohort of 56 women that were grouped by body mass index (BMI) and metabolic syndrome as lean (LE, *n* = 12), overweight (OV, *n* = 10), obese (OB, *n* = 16) or obese with metabolic syndrome (MS, *n* = 16). Plasma KIAA1199 is correlated with fat free mass in OV, OB and MS patients (**a**), total cholesterol (**b**) and triglycerides (**c**) in all the patients. KIAA1199 mRNA levels in abdominal VAT correlated with free fatty acid (**d**), glycerol (**e**) in all patients and leptin mRNA levels in OV, OB and MS groups (**f**). **g** Plasma KIAA1199 correlated with fasting blood glucose in all patients. **h** KIAA1199 mRNA levels in VAT correlated with the values of homeostatic model assessment-insulin resistance (HOMA-R) in all patients. **i** Comparison of KIAA1199 gene expression in VAT in LE, OV, OB and MS four groups’ patients. **j** Gene expression levels of KIAA1199 curated from public databases GOI_GSE20950. The KIAA1199 gene expression in fat tissues from obese patients that are either insulin resistant (*n* = 20) or insulin sensitive (*n* = 19) were compared. **k** UK Biobank based summary statistics of the association between human sequence variations and whole-body fat mass (*n* = 7 637) for chromosome 15. Zoom in to bp 80 280 001–81 990 000 (hg19) surrounding the KIAA1199 locus. The dotted line indicates a *P*-value of 5 × 10^−8^. Analysis of Pearson two-tailed correlation test (*r* = correlation coefficient) was performed using GraphPad Prism software (**a–h**). Statistical difference was determined by one-way ANOVA with Dunnett’s multiple tests (**i**) or two-tailed unpaired Student’s *t*-test between two groups (**j**). The number of independent donors (*n*) in each correlation analysis is described in the results section and in each figure. *P < 0.05, **P < 0.01 and ***P < 0.001

In order to examine the clinical relevance, we examined KIAA1199 plasma levels in a cohort of women that were either lean (LE), overweight (OV), obese (OB) or with metabolic syndrome (MS).^20^ The expressions of KIAA1199 in SAT were positively correlated with body weight (Fig. S20a), BMI (Fig. S20b), fat mass (Fig. S20c), waist circumference (Fig. S20d), hip circumference (Fig. S20e) and sagittal diameter (Fig. S20f), which was particularly significant in OB + MS and MS group. Plasma KIAA1199 levels were negatively correlated with fat free mass in OV, OB and MS women (Fig. 6a), and mostly observed in OB + MS and MS only groups (Fig. S21a). Also, levels of total plasma cholesterol were positively correlated with plasma KIAA1199 levels in the whole group (Fig. 6b) or when examined separately (Fig. S21b). Similarly, plasma KIAA1199 levels were positively correlated with plasma triglycerides levels (Fig. 6c and Fig. S21c). Interestingly, plasma levels of KIAA1199 exhibited age related increase (Fig. S21d). Moreover, we observed KIAA1199 expression levels in abdominal VAT fat biopsies were positively correlated with plasma glycerol (Fig. 6d and Fig. S22a), free fatty acid (Fig. 6e and Fig. S22b); and negatively correlated with leptin expression in VAT (Fig. 6f and Fig. S22c). We did not observe significant correlation with KIAA1199 and adiponectin gene expression (Fig. S22d). KIAA1199 levels in abdominal VAT fat biopsies were also positively correlated with fasting plasma glucose levels (Fig. 6g), and the homeostatic model assessment for insulin resistance (HOMA-IR), an index for insulin resistance (Fig. 6h). Moreover, adipose tissue KIAA1199 expression levels were significantly higher in obese patients with metabolic syndrome (MS) (Fig. 6i).

In addition, we interrogated public gene expression data sets in human fat tissue biopsies^21^ and observed that the expression levels of KIAA1199 in omental and subcutaneous fat were higher in obese patients with insulin resistance (Fig. 6j). Furthermore, we checked if human sequence variations in the vicinity of the KIAA1199 locus in chromosome 15 are linked to phenotypes recorded in the UK Biobank (http://www.nealelab.is/uk-biobank/). We observed that the whole-body fat mass is significantly associated (*P*-value < 5 × 10^−8^), and showed an enrichment of signal near KIAA1199 promoter region (Fig. 6k). These clinical human data corroborate the findings that KIAA1199 is a factor important for obesity, fat formation and energy metabolism.

### KIAA1199 regulates adipocyte differentiation of hBMSC through osteopontin/integrin/AKT/ERK signaling pathways

**Fig. 7:**
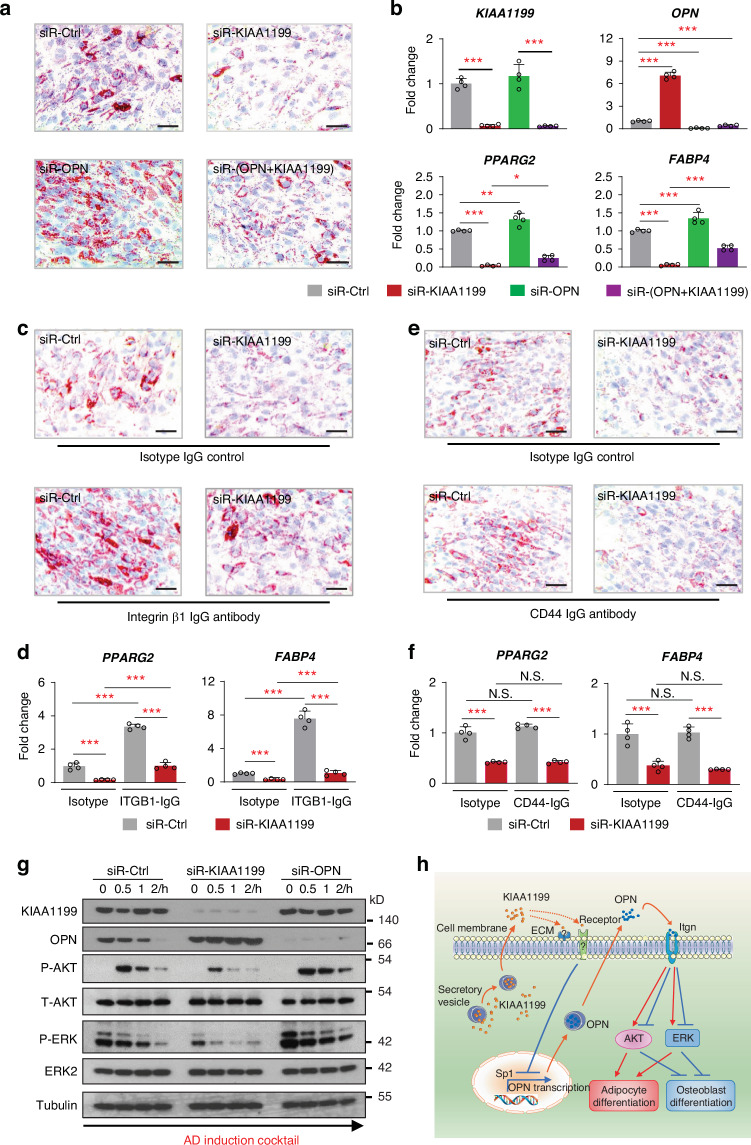
Osteopontin (OPN) / integrin signaling mediates the effects of KIAA1199 on adipocyte differentiation of hBMSC. **a, b** Human bone marrow stromal stem cells (hBMSC) were transfected with specific siRNAs for KIAA1199 (siR-KIAA1199), OPN (siR-OPN), both or control siRNAs (siR-Ctrl), and were induced to adipocyte (AD) differentiation. Mature adipocytes stained by Oil red (**a**) and expression levels of *KIAA1199*, *OPN*, and adipocyte marker genes (*FBAP4* and *PPARG2*) determined by qPCR, *n* = 4 (**b**). **c**–**f** hBMSC were transfected with siR-KIAA1199 or siR-Ctrl. Cells were treated with an integrin β1 (ITGB1) blocking antibody, or an isotype control, or CD44 blocking antibody (each at 10 mg/ml) during AD differentiation. Oil Red staining (**c**, **e**) and gene expression analysis of adipocyte marker genes (*PPARG2, FABP4*) analyzed by qPCR (**d**, **f**), *n* = 4. **g** KIAA1199-OPN axis regulates AD differentiation through ERK and AKT signaling. hBMSC were transfected with siR-KIAA1199 or siR-OPN or siR-Ctrl for 48 h, cells were starved for 6 h, followed by AD induction up to 0, 0.5, 1, 2 h. Western blot analysis for AKT and ERK activation was performed, and beta-tubulin was used as a loading control. **h** A schematic diagram of the possible mechanisms of KIAA1199 interacts with OPN/integrin/AKT/ERK axis on regulating adipogenesis and osteogenesis. Scale bar = 100 µm. All the experiments were performed at least for three times, data are expressed as means ± SD, statistical difference was determined by one-way ANOVA with Dunnett’s multiple tests, *P < 0.05, **P < 0.01 and ***P < 0.001

We have previously reported that osteopontin (OPN) is a KIAA1199 target gene^16^ and deficiency of KIAA1199 enhances osteoblast differentiation of hBMSC through induction of OPN expression.^16^ Similarly, we observed that impaired AD differentiation in KIAA1199 deficient hBMSC (siR-KIAA1199) was associated with higher expression levels of OPN during AD differentiation (Fig. S23a-d). To test whether the causal relationship of OPN and KIAA1199 also applies to AD differentiation, we observed that decreased OPN expression, led to increased expressions of AD markers: PPARγ and FABP4, as well as increased formation of mature adipocytes in hBMSC during AD induction (Fig. 7a, b). Synchronous knockdown of OPN in siR-KIAA1199 cells, rescued the inhibitory effects of KIAA1199 on AD differentiation (Fig. 7a, b). Since OPN is an extracellular matrix protein that mediates its effects through binding to integrin β1 or CD44,^22–24^ to further investigate the mechanism of OPN regulation of AD differentiation, hBMSC were treated with an integrin β1 blocking antibody and we observed enhanced AD differentiation and abolishing the impaired AD differentiation in siRNA-KIAA1199 (Fig. 7c, d). On the other hand, similar experiments employing neutralizing antibody of CD44 did not affect AD differentiation (Fig. 7e, f).

In follow up studies, we examined the signaling pathways activated during AD differentiation by Western blot analysis. siRNA-KIAA1199 hBMSC exhibited reduced levels of AKT and ERK phosphorylation (P-AKT, P-ERK) following AD induction (Fig. 7g and Fig. S23e); while knocking down of OPN (siR-OPN) led to opposite effects with enhanced of P-AKT and P-ERK (Fig. 7g and Fig. S23f). Taken together the molecular mechanism that we reported for KIAA1199 effects on bone formation and osteoblast (OB) differentiation,^16^ applies to fat formation and AD differentiation where the OPN/integrin signaling mediates the regulatory effects of KIAA1199 on lineage differentiation of hBMSC and involve down-stream effectors of AKT and ERK signaling pathways. Interestingly, this signaling pathway functions in a reciprocal fashion in AD versus OB differentiation as illustrated in the proposed working model (Fig. 7h).

## Discussion

Bone remodeling is a life-long dynamic regenerative and energy demanding process. In the past decade, a number of factors regulating bone remodeling have been identified to play an endocrine functions, coordinating the energy requirement for bone remodeling and bone formation with the whole-body energy homeostasis.^1^ In the current study, we report that KIAA1199, a secreted factor produced by bone and BMSCs that regulates bone formation and regeneration,^16^ exerts novel regulatory effects on BMAT and systemic fat formation, as well as whole-body energy metabolism.

We have observed that KIAA1199 is expressed in human bone tissue biopsies in marrow stromal cells around mature bone marrow adipocytes and is absent in mature adipocytes. This pattern of expression is similar to what we described previously that KIAA1199 that is expressed in osteoprogenitor cells and not mature osteoblasts.^16^ It is plausible that such anatomical location is ideal for recruitment of progenitor cells for future bone marrow adipocyte formation and corroborate our in vitro data showing expression of KIAA1199 in adipocyte progenitors and its low expression in mature adipocytes. Marrow adipose tissue (BMAT) is increasingly recognized as an important fat depot that participates in whole-body energy homeostasis and constitutes around 10% of total body fat and 50%–70% of total bone marrow volume.^25^ Under physiological conditions, BMAT is thought to provide energy needed for of bone remodeling and thus exhibits significant changes in metabolic bone diseases.^25^ We demonstrate that KIAA1199 acts locally in the bone microenvironment by regulating BMAT differentiation in vitro and enhances BMAT formation in vivo as its deficiency is associated with strong reduction in BMAT volume. Interestingly, decreased BMAT mass is associated with reduced extramedullary fat mass in SAT and VAT depots, and improved metabolic health under both normal feeding and during HFD feeding by increasing tissue insulin sensitivity, and associated with reduction of fasting glucose and plasma lipids. These results suggest that KIAA1199, a factor secreted by bone and BMSCs, regulates BMAT formation through interaction with whole body energy metabolism.

Formation of BMAT requires the availability of adipocyte progenitor cells and the presence of macronutrient substrate need for lipogenesis. It is plausible that KIAA1199 regulates BMSC by being permissive for adipocyte progenitor differentiation (present study) and inhibitor of the osteoblast progenitor differentiation,^16^ thus acts as a molecular switch regulating osteoprogenitor and adipoprogenitor populations in the local bone microenvironment. On the other hand, KIAA1199 through decreasing insulin sensitivity in target organs on the systemic levels, enables substrate availability for BMAT and extramedullary fat formation.

There are several possible molecular mechanisms underlying KIAA1199 effects on adipocyte formation. KIAA1199 is known to degrade HA,^11^ which is a ligand for CD44 that regulates adipocyte cell formation.^26^ Another plausible mechanism is the observed inhibitory effects of KIAA1199 on osteopontin (OPN) and its interaction with its cognate receptor ITGB1.^16^ OPN is also known for its effects on extramedullary AD differentiation.^27^ Interestingly, OPN knockout (*OPN*^*−/−*^) mice have been reported to be protected from obesity, insulin resistance, adipose tissue inflammation, and hepatic steatosis during HFD feeding,^27–29^ which is similar phenotype to what we observed in KIAA1199 KO mice. While the metabolic effects of OPN deficiency might be mediated through its CD44 receptors and manifested systemically,^30^ our observed effects caused by KIAA1199-OPN interaction are mediated through ITGB1 receptor and manifested in bone microenvironment, suggesting the presence of correlated mechanisms regulating the size of extramedullary versus medullary adipose tissue.

A number of intracellular signaling pathways are involved in regulating BMSC fate to AD including ECM-Integrin, BMP, Wnt, Hedgehogs, Notch, and FGFs signaling as well as PI3K-AKT and MAPK-ERK1/2 signaling.^31–33^ We observed that KIAA1199 effects on AD differentiation were associated with changes in P-AKT and P-ERK signaling downstream of its effects of OPN/integrin signaling. Interestingly, ERK signaling has been proposed to initiate the conversion of the adipoprogenitor cells into AD.^34^ It is plausible that down regulation of KIAA1199 expression is required to allow the progression of AD formation and that ERK signaling is marker of this transition. In addition, since AKT and ERK are also down-stream effectors of insulin signaling, our finding provides an additional possible molecular mechanism for the ability of KIAA1199 to interact with insulin signaling in extramedullary tissues. As mentioned above, deficiency of KIAA1199 in hBMSC acts in reciprocal fashion in regulating osteogenesis and adipogenesis of BMSC, suggesting that KIAA1199/OPN/integrin/AKT/ERK signaling acts as a dual switch for BMSC lineage fate determination in local bone microenvironment.

Some studies have reported a positive association between circulating levels of KIAA1199 and inflammatory diseases and clinical prognosis in several cancer types.^11,13,35–42^ We have observed that expression level of KIAA1199 is a possible biomarker for metabolic health as KIAA1199 tissue expression and/or plasma levels exhibit positive correlation with clinical characteristics of obesity and metabolic syndrome, which are characterized by the presence of a chronic low-grade inflammation. Interestingly previous studies have reported that increased expression level of KIAA1199 by proinflammatory cytokines through NF-kB signaling.^35,43–45^ The concept that KIAA1199 is a mediator of chronic ‘sterile’ inflammation may explain partly the observed biological effects of KIAA1199 during HFD feeding and requires additional studies for its potential clinical translation.

We have recently reported that KIAA1199 regulates bone formation during adult bone remodeling and its absence leads to increased bone mass and enhanced bone regeneration following bone fracture, and that it is expressed at a higher level in osteoporosis patients^16^ suggesting that the biological role of KIAA1199 extends beyond development to regulation of adult tissue homeostasis and its relevance to the pathophysiology of human aging. The present study corroborates this hypothesis as we reported that KIAA1199 expression and secretion regulate BMAT formation and correlate with insulin sensitivity, glucose and lipid metabolism and its plasma levels are increased with donor age. Is there a relationship between these observations? Our data corroborate the findings provided by an increasing number of investigators who identified factors that link bone remodeling, BMAT formation and whole-body energy metabolism. Among these, osteocalcin, lipocalin, leptin, adiponectin^1,46^ and in the current study KIAA1199. A common theme for all these factors is that they exert dual regulatory role: in bone microenvironment and at the systemic levels. These new discoveries as well as our findings suggest a possible clinical translation by targeting KIAA1199 with monoclonal antibody (mAb) or small molecule chemical inhibitor, for treatment of conditions of bone fragility and associated metabolic diseases of obesity and insulin resistance.

## Materials and methods

### Ethics statement

Permissions to perform all animal works and protocols in KIAA1199 knockout and corresponding wild-type mice in this study were granted by the Danish Animal Experiments Inspectorate (No. 2017-15-0201-01210). All the related measurements and surgeries on animals were performed in a rodent-dedicated animal center in Odense University Hospital complied with all relevant European ethical regulations for vertebrate animal research as description before^16^ Informed patient consent for generation and subsequent use of the human samples in this study were given by the Scientific Ethics Committee at the Charles University in Prague, Czech Republic.

### Cell culture

A telomerized human bone marrow stromal stem cell (hBMSC-TERT) was established from health male bone marrow hBMSC through overexpression of human telomerase reverse transcriptase gene and has proved the ‘stemness’ characteristics,^47^ the stable cell line was used as hBMSCs in this study. hBMSC were cultured and passaged in basal culture medium with Minimum Essential Medium (MEM) with 10% fetal bovine serum (FBS) and 1% penicillin-streptomycin (P/S). Mouse primary bone marrow MSC (mBMSC) were isolated from mouse tibia and femur bone marrow, and mouse primary adipose-derived MSC (mADMSC) were isolated from mouse subcutaneous adipose tissue. Briefly, cells were collected from digested bone or fat tissues and filtered through 40 µm nylon mesh, then cultured in Minimum Essential Medium (MEM) with 20% FBS, 1 mmol/L pyruvate, 1x Non-Essential Amino Acids and 1% P/S. Half of the medium was replaced every 3 days. At confluence, cells were harvested and passaged. Cells were incubated in 5% CO_2_ incubators at 37 °C. All cell culturing reagents were purchased from Life Technologies (Taastrup, Denmark).

### Induction of adipocyte (AD) differentiation

hBMSC or mBMSC or ADMSC were cultured to reach confluence and the cells were then induced to adipocyte differentiation by adipocyte induction mediums consisting of MEM medium supplemented with 10% FBS, 10% horse serum, 1% P/S (Life Technologies, Taastrup, Denmark), 100 nmol/L dexamethasone, 0.45 mmol/L isobutyl methyl xanthine (IBMX), 3 μg/mL insulin and 1 μmol/L rosiglitazone (BRL49653) (Sigma, Copenhagen, Denmark). Differentiation induction medium was changed every two days, the induction of differentiation lasted for 7–14 days according to the requirements for different measurements.

### Oil Red staining

Oil Red O staining was performed to measure the formation of lipid droplet-filled mature adipocytes. Cells were washed with PBS, fixed with 4% paraformaldehyde for 10 min and then incubated with 0.45 μmol/L Oil Red O in isopropanol solution (Sigma, Copenhagen, Denmark) for 1 h at room temperature and washed by water. The photomicrograph images were acquired using invented phased-contrast microscope (Zeiss, Oberkochen, Germany).

### Quantitative real-time qPCR

RNA was isolated by TRIzol^TM^ according to the manufacturer’s instructions (Thermo-Fisher Scientific, Roskilde, Denmark). The first strand complementary DNA was synthesized from 1 µg total RNA by Revert aid cDNA kit (Sigma, Copenhagen, Denmark). The PCR products were visualized in real-time using iQ^TM^ SYBR Green I Supermix (Bio-Rad) and an iCycle instrument (Bio-Rad) using standard curve protocols, normalized to the geometric mean of the reference genes. Primer sequences for the genes tested are shown in Table S1.

### Cell transfection

The transfection in cells was performed at 70%–90% confluence using Lipofectamine^TM^ 2000 (Life Technologies, Taastrup, Denmark) with siRNAs according to the manufacturer’s recommendations. The siRNAs for specific genes (siR-KIAA1199: s32900, s32899, s32901; OPN: s13377, s13375, s13376) or control non-targeting siRNA (4404021, 4390846) were all purchased from Silencer® Selector siRNA library from Thermo-Fisher Scientific (Roskilde, Denmark). The conditioned medium (CM) from the KIAA1199 overexpression hBMSC and corresponding vector transfected cells was performed as described before.^15^

### Western blot analysis

The cells were washed in cold PBS and lysed in RIPA buffer (Thermo-Fisher Scientific, Roskilde, Denmark) supplemented with protease inhibitors (Roche, Hvidovre, Denmark) for more than 30 min. Samples were centrifuged at 13 000 r/min, 4 °C for 10 min. Protein concentrations were determined with a BCA kit (Thermo-Fisher Scientific, Roskilde, Denmark), and equal amounts (30 µg) of proteins were loaded on a polyacrylamide gel (Thermo-Fisher Scientific, Roskilde, Denmark). Blotted nitrocellulose membranes were incubated overnight with primary antibody at 4 °C and were developed after 1 h incubation with secondary anti-rabbit horseradish peroxidase-conjugated antibody (Santa Cruz Biotechnology, USA) using an ECL Western blotting kit (GE Healthcare, USA) and Kodak films. Antibodies for KIAA1199 (#HPA044676), for alpha-tubulin (#T9026, clone DM1A) and for beta-actin (#A2066) were from Sigma, Søborg, Denmark, and other antibodies used in the study (phos-AKT, #4051 clone587F11, AKT: #9272, phos-ERK: #4695, clone137F5, ERK: #9101) were purchased from Cell Signaling Technology (Herlev, Denmark).

### *KIAA1199*^*−/−*^ mice generated by CRISPR/Cas9 technology

KIAA1199 knockout (*KIAA1199*^*−/−*^, KO) mice were generated by CRISPR/Cas9 technology on C57BL/6 J background in our lab as described before.^16^ Mice were fed ad libitum normal chow diet with 4.5% fat, 14.5% protein, 60% carbohydrate (Lantmännen; Germany, # R70) or high-fat diet with 60% fat, 20% protein, 20% carbohydrate (New Brunswick, NJ, USA, Research Diet #D12492) at 8 weeks of age for 20 weeks. Animals were bred and housed under standard conditions (21 °C, 55% relative humidity) on a 12-h light/dark cycle. All experimental procedures were approved by the Danish Animal Ethical Experiments Inspectorate (No. 2017-15-0201-01210).

### Body composition measurements

Mice total fat mass (g), fat mass ratio (%), lean mass (g) and lean mass ratio (%) were determined by dual-energy X-ray absorptiometry (DEXA) with a Lunar PIXImus2® densitometer (Version 1.44, Lunar Corporation, Madison, WI, USA). DEXA scans were performed every month after sedating mice with isoflurane starting at 8 weeks of age and ending at 30 weeks after feeding controls began. Body weights of mice and final dissected tissues weights were measured by using an XL-300 balance (Denver Instrument GmbH, Gottingen, Germany).

### Contrast-enhanced µCT (CE-µCT) scanning

CE-µCT was performed to visualize, apart from the bone, the bone marrow adipose tissue (BMAT). Briefly, the tibias were fixed for 72 h in paraformaldehyde (4%) and were transferred to PBS. Then, they were put in a Hafnium-substituted Wells Dawson polyoxometalate (Hf-POM)-based staining agent solution (35 mg of Hf-POM per 1 mL PBS)^19^ for 48 h prior to CE-µCT imaging. Similar acquisition and reconstruction settings were applied as described previously.^19^ In brief, a Phoenix NanoTom M (GE Measurement and Control solutions, Germany) was used for image acquisition (voltage of 70 kV and a current of 60 µA, 0.2 mm filter of aluminum filter). Scanning was performed at a 2 µm isotropic voxel size. For analyses of BMAT, we drew volumes of interest around the trabecular bone region in the proximal metaphysis starting directly from the end of tibiae and covering a height of 5 mm distally. We analyzed the following structural parameters using CTAn (Bruker MicroCT, Kontich, Belgium): marrow adipose volume (mm^3^), marrow adipose volume / corresponding marrow space (V/V).

### Enzyme-linked immunosorbent assay (ELISA) and chemical quantitation assay

Human plasma and mouse fasting plasma were harvested as the approved application by the Scientific Ethics Committee. Quantitative determinations of different factors were measured by the corresponding ELISA kits or activity assay kits following the manufacture manuals (hKIAA1199: from Antibody Online, #ABIN457025; mOPN: from Abcam, # ab100734; cholesterol: from Abcam, #ab65390; triglyceride, free fatty acid: from Sigma, #TR0100, #MAK044; adiponectin: from ThermoFisher, #KMP0041, glycerol: from Zen Bio, #SGA-1).

### Blood glucose measurement, glucose tolerance test (GTT), insulin tolerance test (ITT) and glucose induced insulin secretion (GSIS)

Blood glucose was measured on blood from the mouse tail. Mice on ND and HFD were fasted for 16 h or 4 h before subject to GTT or ITT, respectively. Mice were measured starting plasma glucose (0 min), then intra-peritoneal injected of glucose (1 mg/g) or insulin (0.5 mU/g), blood glucose was measured on tail blood using a CONTOUR® Glucometer at 15, 30, 60, 90 and 120 min after injection, respectively.

For glucose-stimulated insulin secretion (GSIS), overnight-fasted mice were intra-peritoneal injected with glucose (2 mg/g body weight), and tail blood were collected at 0, 2, 5, 15 and 30 min, plasma insulin was measured using mouse ultrasensitive insulin ELISA Kit (ThermoFisher, #EMINS).

### Histology and immunohistochemistry

Mouse tissues were fixed with 4% formalin for 1 days then change to phosphate buffered saline (PBS). Tissues were embedded in paraffin and sections were used for hematoxylin and eosin (H&E) staining or immunostaining. In brief, paraffin tissue sections were deparaffinized, dehydrated, and heat treated before incubation with primary antibody against insulin (DAKO, #A0564) and F4/80 (Bio-Rad, #MCA497G) followed by secondary antibody, chromogen visualization and counterstaining with hematoxylin.

### Measurements on clinical samples from patients and bioinformatic analysis in public microarray database

Fifty-six Caucasian women excluded from malignancy, inflammatory conditions (based on clinical and laboratory findings), congestive heart failure, known coronary heart disease, known endocrinopathies, chronic liver or kidney disease and psychiatric disorders were grouped into Lean (LE), overweight (OV), obese (OB) and obesity with metabolic syndrome (MS) according to their BMI and clinical investigation from Charles University in Prague, Czech.^20^ Their body weight had been stable for 3 months prior to the examination. Plasma was collected and adipose tissues were harvested from abdominal surgery (laparoscopic or laparotomic cholecystectomy, hysterectomy and gastric banding). The informed consent was obtained from each patient before the study. The study was performed according to the Declaration of Helsinki protocols and was approved by Ethical Committee of the Third Faculty of Medicine, Charles University in Prague, Czech.

Bioinformatics’ analysis was performed on publicly available RNA-seq and Single-cell-sequence database from GOI_GSE20950^17^ and chromatin interaction data,^18^ and microarray databases GOI_GSE20950 from omental adipose tissue and subcutaneous adipose tissues in morbidly obese patients with insulin sensitive or insulin resistant.^21^

### Statistical analysis

All the data were collected from at least two independent experiments and presented as the mean and standard deviation (SD). Normality test was applied to all data to test data distribution. Two tailed student’s *t*-test (a parametric or non-parametric) was used to assess differences between two groups’ comparisons according to the data distribution. Two tailed student’s *t*-test (a parametric or non-parametric) was used to assess differences between two groups’ comparisons according to the data distribution. One-way ANOVA was used for more than two groups’ comparisons with one condition, ANOVA analysis was followed by post hoc test for multiple comparison. The correlation statistical analyses between variables were performed using the Pearson two-tailed correlation test (*r* = correlation coefficient). Analysis of correlation was performed using GraphPad Prism software. The number of independent samples (*n*) in each analysis is described in each figure or figure legend. Statistical significance was considered when *P* < 0.05.

## Supplementary information

Supplemental materials

## Data Availability

The data that support the findings of this study are available from the corresponding author upon request.
