# Supplementary material for: KIAA1199 (CEMIP) regulates adipogenesis and whole-body energy metabolism
Source: Bone Res. 2025 Apr 2;13:43. doi: 10.1038/s41413-025-00415-2 (PMC11961601; doi:10.1038/s41413-025-00415-2)
Supplement: Supplementary file 1 — Supplemental materials [file 41413_2025_415_MOESM1_ESM.pdf]

Title: KIAA1199 (CEMIP) regulates adipogenesis and whole-body energy metabolism

Supplementary information contents:

1. **Supplementary Material and Methods**
2. **Supplementary Figures: S1-S23**
3. **Supplementary Table 1 (**PCR primers for different genes used in the real time qPCR in the study)

Supplementary Materials and Methods:

In situ hybridization

The expression of KIAA1199 mRNA in human bone marrow was assessed using *in situ* hybridization, following a modified version of the RNAScope 2.5 High-Definition assay (310035, Advanced Cell Diagnostics [ACD], Hayward, CA, USA). Paraffin-embedded, 3.5-μm-thick sections were prepared from decalcified 3-mm iliac crest needle biopsies obtained from four human bone samples. Tissue sections were pretreated and hybridized with a custom-designed probe containing 20 ZZ-pairs (Probe-Hs-CEMIP, 449811, ACD), targeting KIAA1199 (region 1934–2896 of NM_001293298.1). Ethical approval for the study was obtained from the Danish Regional Committee on Biomedical Research Ethics (S-20070121).

Bioinformatic analysis of chromatin interaction for KIAA1199

Bioinformatics analysis was conducted using publicly available RNA-seq and single-cell sequencing datasets (GSE2095017)[1] along with chromatin interaction data[2]. Previously

established enhancer activity maps generated during human adipocyte differentiation were utilized to identify regulatory regions upstream of the KIAA1199 gene promoter on chromosome 15. These regions were analyzed in the context of KIAA1199 expression suppression during adipogenesis at days 0, 1, and 7. Enhancer-centered chromatin interaction profiles of chromosome 15 were generated using enhancer capture Hi-C, the interactions between the enhancer region and the KIAA1199 gene promoter were further examined following adipogenic stimulation at the specified time points (days 0, 1, and 7).

Mouse breeding and feeding regimes, indirect calorimetry

All animal experimental procedures were approved by the Danish Animal Ethical committee regarding the protection of animals used for experimental and other scientific purposes (No. 2017-15-0201-01210). Mice were housed three or four per cage and fed by regular chow and water *ad libitum* under standard conditions (21°C, 55 % relative humidity) on a 12-hour light / dark cycle at the animal facility of University of South Denmark.

KIAA1199 knockout (KO) mice and wild-type (WT) mice (5-6 weeks old) obtained from heterozygous mating were fed *an libitum* normal diet (ND; Lantmännen; containing Kcal %: protein 14.5 %, carbohydrates 60 %, and fat 4.5 %) or 60 kcal % high-fat diet (HFD; Research Diet D12492, containing Kcal %: protein 20 %, carbohydrates 20 %, and fat 60 %) in which fat content was from soybean oil and lard) at 5-6 weeks of age for a period of 20 or 28 weeks. Separate cohorts of animals on ND and HFD were used for metabolic cage analysis using a 16-chamber indirect calorimetry system (PhenoMaster; TSE Systems, Bad Homburg, Germany). Mice were individually housed in the chambers for 7 days; the first 3 days were used for acclimatization, and data were analyzed from the last 4 days. Oxygen consumption rate, carbon dioxide production rate, feeding, and total locomotive activity were measured concurrently for each mouse.

Human KIAA1199 protein purification and silver staining

The KIAA1199 (Gene ID: NM_001293298.1) expression plasmid with an HA-tag was constructed using the pLVX-mCMV-ZsGreen-Puro vector (BioWit, Shenzhen, China). Human bone marrow stromal cells (hBMSCs) and HEK-293T cells were infected with the KIAA1199 overexpression virus or the corresponding empty vector, stable cell lines were established. Conditional medium (CM) was collected as previously described[3]. The CM was concentrated using Amicon™ Ultra-15 Centrifugal Filter Units with a 3 kDa molecular weight cutoff (MilliporeSigma, Thermo Fisher Scientific, Shanghai, China). KIAA1199 protein was purified from the 25-fold concentrated CM. Purification involved dialysis using a 100 kDa dialysis membrane in deionized water for 24 hours with water changes every 4 hours, followed by binding to BeyoMag™ anti-HA magnetic beads (Beyotime, Shanghai, China). The beads were washed five times with PBST buffer, and the protein was eluted. Purified proteins were resolved via PAGE and visualized using the Silver Staining Kit (Solarbio Science & Technology, Beijing, China) according to the manufacturer's instructions. After staining, the gel was washed with distilled water, and protein bands appeared as dark brown against a light background.

Reference:

1 Rauch, A. *et al.* Osteogenesis depends on commissioning of a network of stem cell transcription factors that act as repressors of adipogenesis. *Nat Genet* **51**, 716-727, doi:10.1038/s41588-019-0359-1 (2019).

2 Madsen, J. G. S. *et al.* Highly interconnected enhancer communities control lineage-determining genes in human mesenchymal stem cells. *Nat Genet* **52**, 1227-1238, doi:10.1038/s41588-020-0709-z (2020).

3 Chen, L., Shi, K., Andersen, T. L., Qiu, W. & Kassem, M. KIAA1199 is a secreted molecule that enhances osteoblastic stem cell migration and recruitment. *Cell Death Dis* **10**, 126, doi:10.1038/s41419-018-1202-9 (2019).

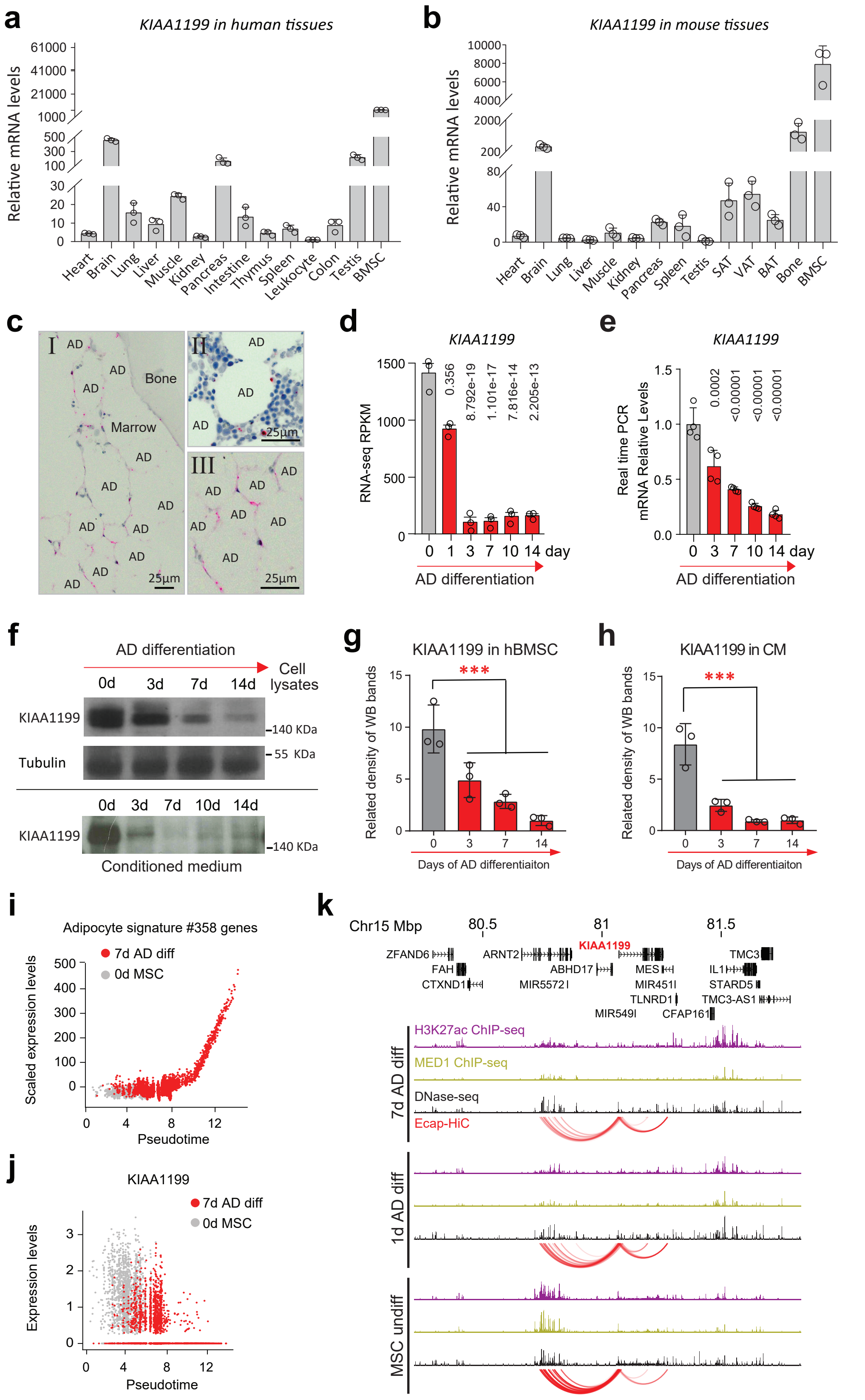

Fig. S1. KIAA1199 highly expresses in bone and bone marrow stromal stem cells, and its expression and secretion are significantly reduced during adipocyte (AD)

differentiation in hBMSCs. (a) Relative expression levels of KIAA1199 mRNA in different human tissue cDNAs (Human MTC Panel, Takara Bio Europe) were measured by qPCR, GAPDH as reference gene, n ≥ 3. **(b)** Relative expression levels of KIAA1199 mRNA in different mouse tissues by real time qPCR. 36B4 gene as reference gene. SAT: subcutaneous adipocyte tissue, VAT: visceral adipocyte tissue, BAT: brown adipocyte tissue, Bone: tibiae with bone marrow. BMSC: bone marrow stromal stem cells, n ≥ 3. **(c)** In situ hybridization (ISH) analysis of KIAA1199 mRNA expression (red dots) in human bone biopsies, with bone marrow cells and bone marrow adipocytes, scale bar = 25 µm, n = 3. **(d-h)** The expression of KIAA1199 were analyzed in hBMSC during AD differentiation at day (d) 0, 3, 7 and 14 by RNA-seq **(d)**, real time qPCR **(e)** and Western blot analysis in cell lysates **(f, g)** and cell conditioned medium **(f, h). (i-j)** Single-cell-sequence analysis of KIAA1199 expression at day (d) 0 and 7 during AD differentiation in hBMSC. Single cell RNA-seq based expression of KIAA1199 (log scale) in bone marrow derived TERT immortalized MSCs prior (n = 1775) to and after 7 days (n = 7012) of adipogenic stimulation. The cells were grouped as grey (0 d) and red spots (7 d) **(i)**, the expressions of KIAA1199 ((log scale) were analyzed in these two groups **(j)**. **(k)** Genome browser screen shot of the loci represented in for chromosome 15, zoom in to bp 80280001 – 81990000 (hg19) surrounding the KIAA1199 locus. Showing DNase-seq, MED1 and H3K27ac ChIP-seq read density and enhancer capture HiC based interaction frequencies of putative regulatory genomic regions with the KIAA1199 promoter in AD differentiating of hBMSC (combined tracks from two independent experiments).

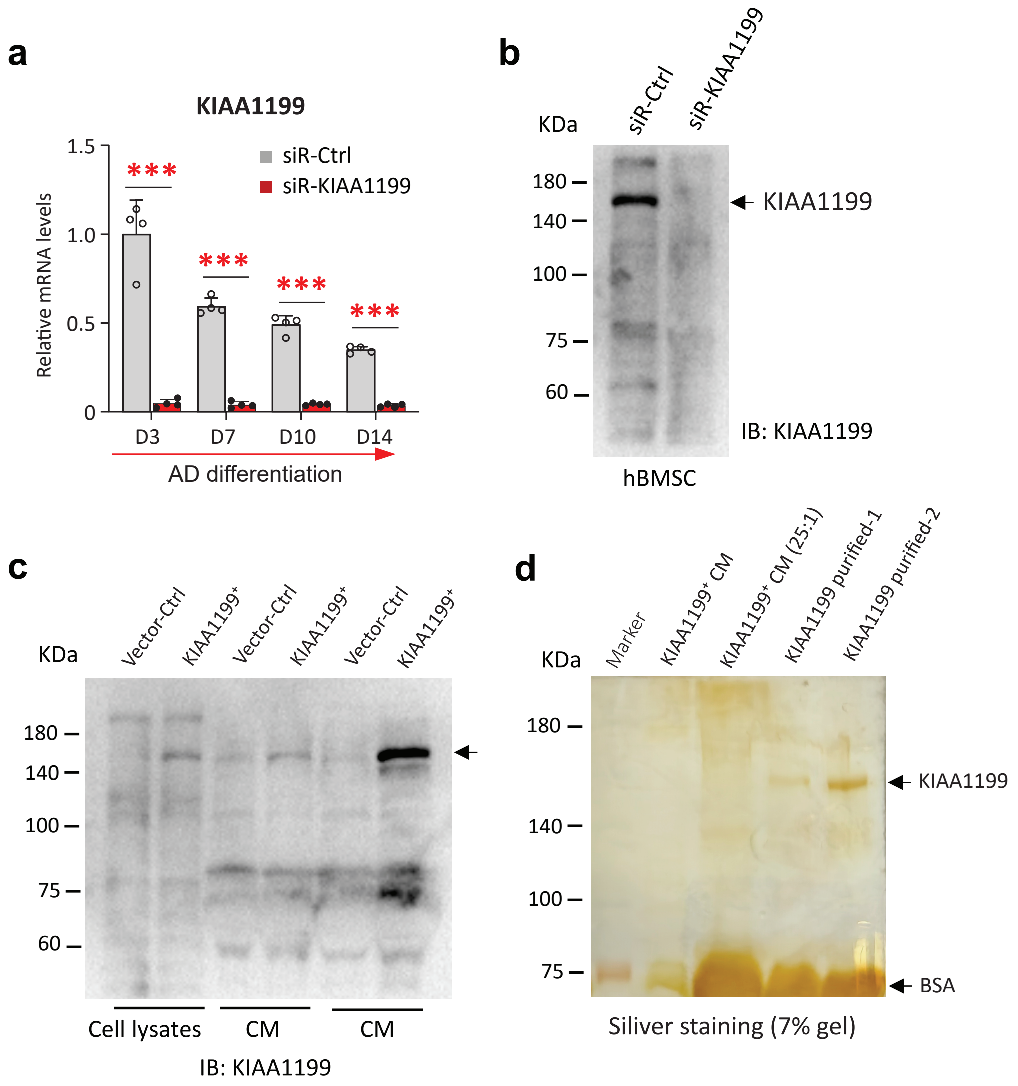

Fig. S2. Examination of KIAA1199 expression, secretion and purification in siRNA knockdown or KIAA1199 overexpression hBMSCs and conditional medium. (a) Human bone mesenchymal stem cells (hBMSC) were cultured and transfected by specific siRNAs of hKIAA1199, then induced to adipocyte differentiation (AD) for 14 days. The expression of KIAA1199 were traced by qRT-PCR in KIAA1199 deficient hBMSC (siR-KIAA1199) and control hBMSC (siR-Ctrl) on Day 3, 7, 10 and 14 during AD differentiation. **(b)** Western blot detection of KIAA1199 expressions in KIAA1199 deficient hBMSC (siR-KIAA1199) and control (siR-Ctrl) hBMSC after 48 hours transfection. **(c)** Western blot of KIAA1199 expressions and secretion in stable transfection Vector-Ctrl and KIAA1199 overexpression ($KIAA1199^{+}$) hBMSC cell lysates and conditional medium (CM, 25:1 concentrated). **(d)** Sliver staining of proteins in KIAA1199 overexpression hBMSCs conditional medium (CM) and the purified KIAA1199 protein from the CM. Data are expressed as means ± SD, the comparison between two groups is performed by two-tailed unpaired Student's t-test, ***P <0.001.

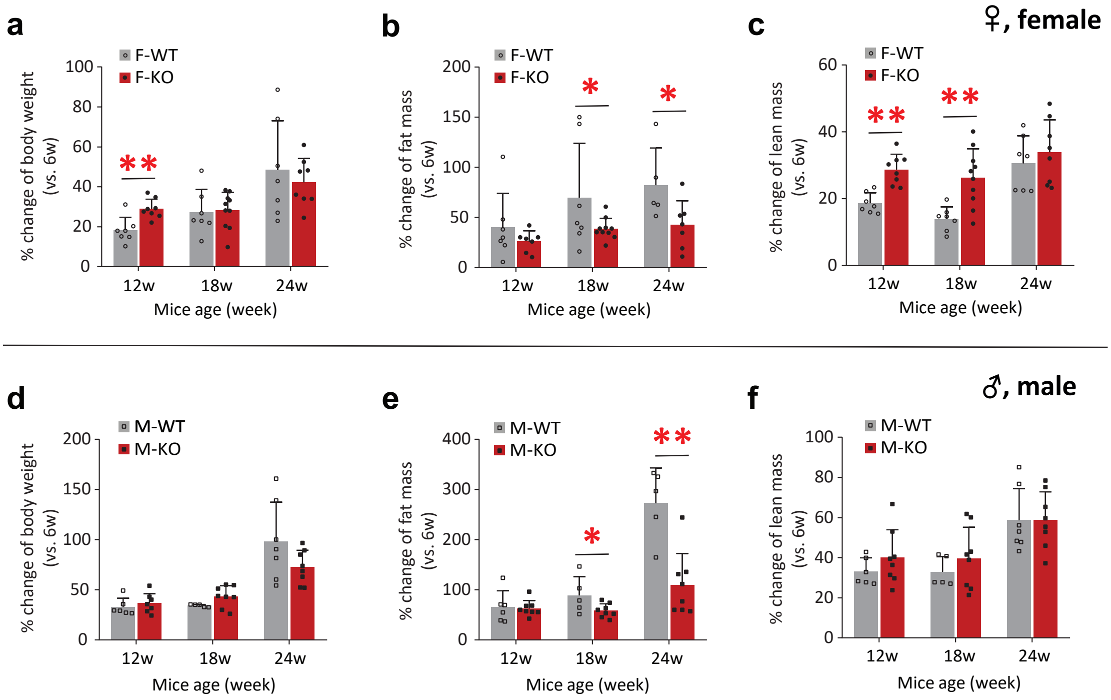

Fig. S3. KIAA1199 knockout (KO) mice significantly reduce fat formation, and increase lean mass. The percentage change of body weight **(a, d)**, lean mass **(b, e)** and fat mass **(c, f)** in female **(a-c)** and male **(d-f)** under normal diet at age of 12, 18 and 24 weeks compare to the 6 weeks when measurement started. n = 6-10 /group. Data are expressed as means ± SD, the comparison between WT and KO groups are performed by two-tailed unpaired Student's t-test. *P < 0.05, **P < 0.01.

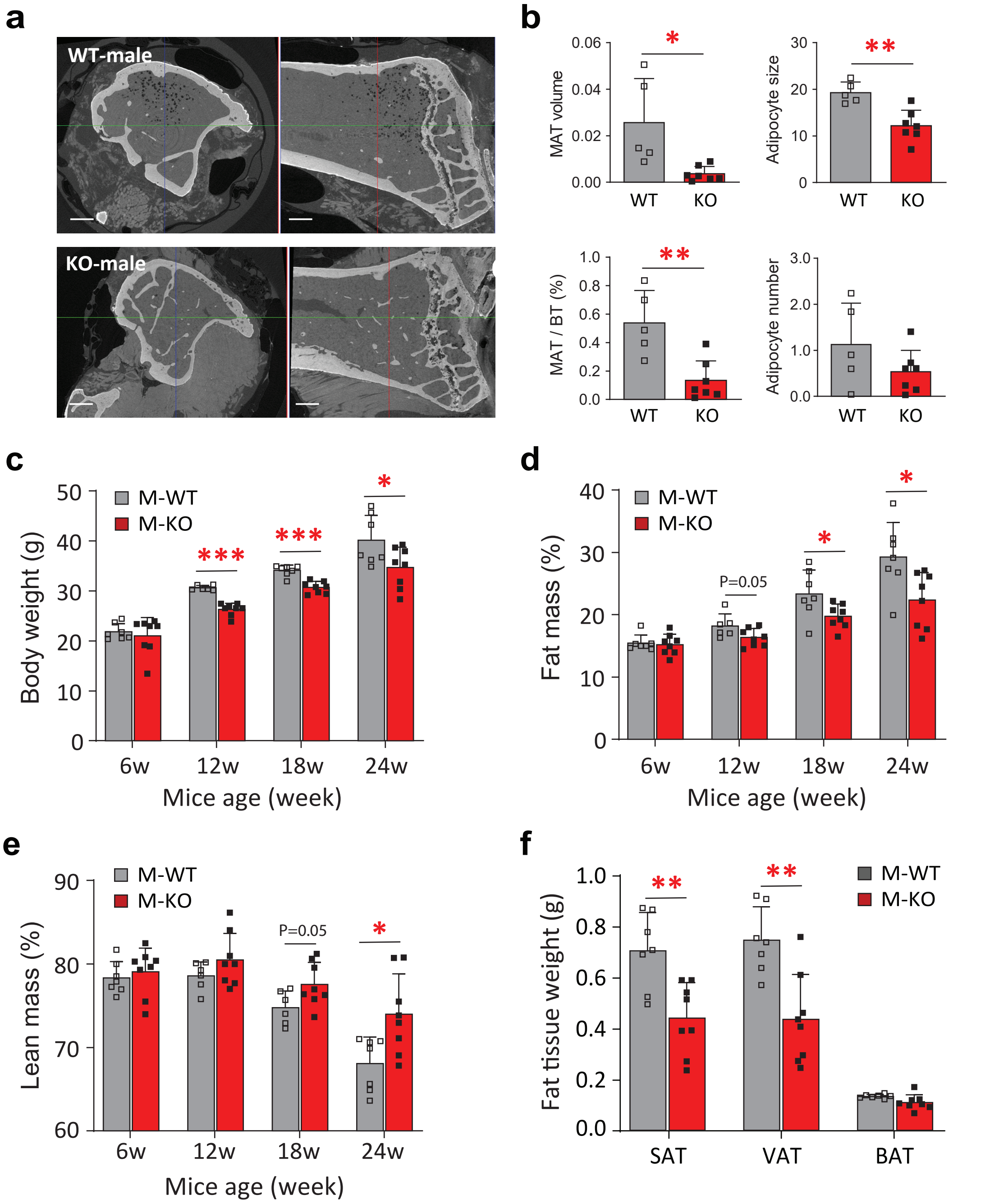

Fig. S4. Characterization of the bone marrow fat and body fat in male KIAA1199 knock-out (KO) and corresponding wild-type (WT) mice. (a-b) The tibiae from matched WT and KIAA1199 KO male mice were stained and scanned by CE-microCT as described in 'Material and Methods', the representative scan images were shown (a), the bone marrow fat (MAT) volume (MAT volume), percentage in the bone marrow space (MAT/BM, v/v), MAT adipocyte size and adipocyte number were quantitated **(b)**. **(c-e)** Body weight, fat mass (%) and lean mass (%) of WT and KO male mice were measured by DEXA scan from the age 6-24 weeks. **(f)** Fat tissue samples (SAT: subcutaneous adipocyte tissue, VAT: visceral adipocyte tissue, BAT: brown adipocyte tissue) from the corresponding sites in mice were collected and weighted when mice were scarified. WT: n = 7; KO: n = 8. Experiments were repeated in three independent cohorts. Data are expressed as means ± SD, the comparison between WT and KO groups are performed by two-tailed unpaired Student's t-test. *P < 0.05, **P < 0.01, ***P <0.001.

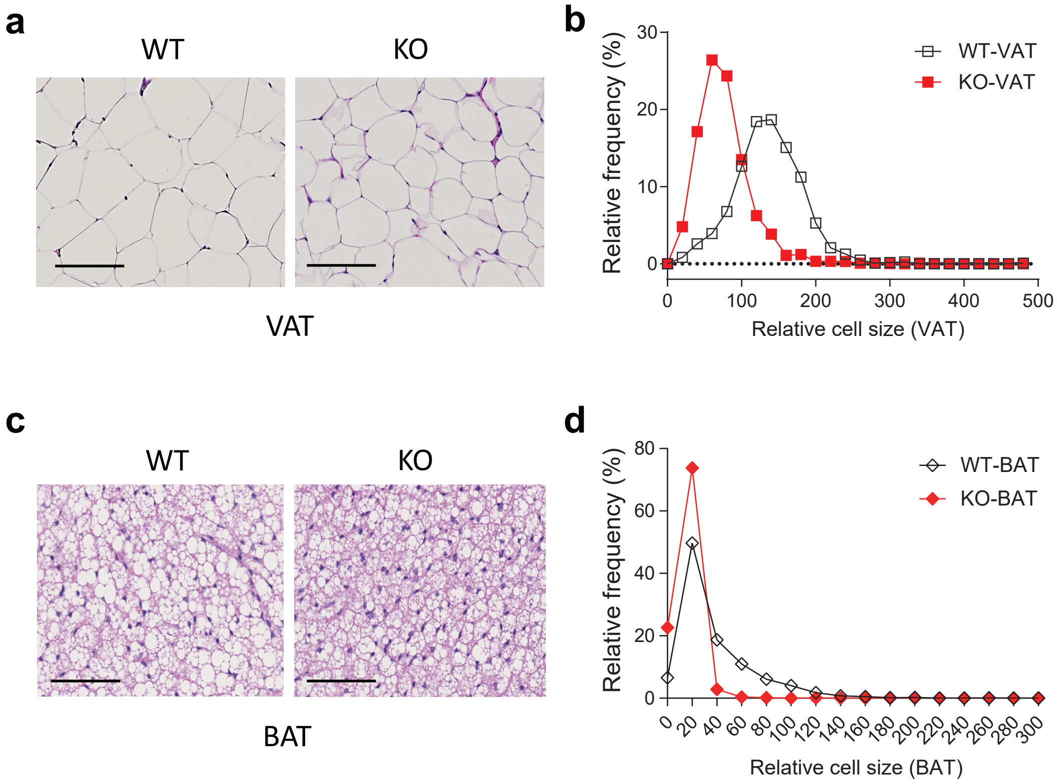

Fig. S5. Characterization of the visceral adipose tissue (VAT) and brown adipose tissue (BAT) from KIAA1199 KO and WT under normal diet. Photomicrographs of H&E staining histological sections of VAT **(a)** and BAT **(c)** from KIAA1199 KO and WT under normal diet and adipocyte size were measured by ImageJ (Adiposoft) and the cell size frequency were analyzed in GraphPad 7 **(b&d)**. Scale bar = 100 µm, n = 7.

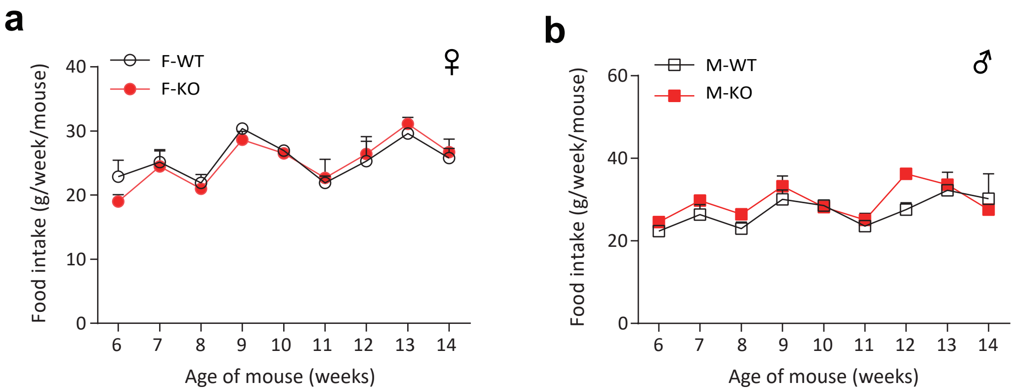

Fig. S6. KIAA1199 KO mice have similar food intakes as control WT mice. Food intakes were traced in female **(a)** and male **(b)** at age of 6-14 weeks. n = 6-10 /group, Data are expressed as means ± SD, the comparison between WT and KO groups are performed by two-tailed unpaired Student's t-test.

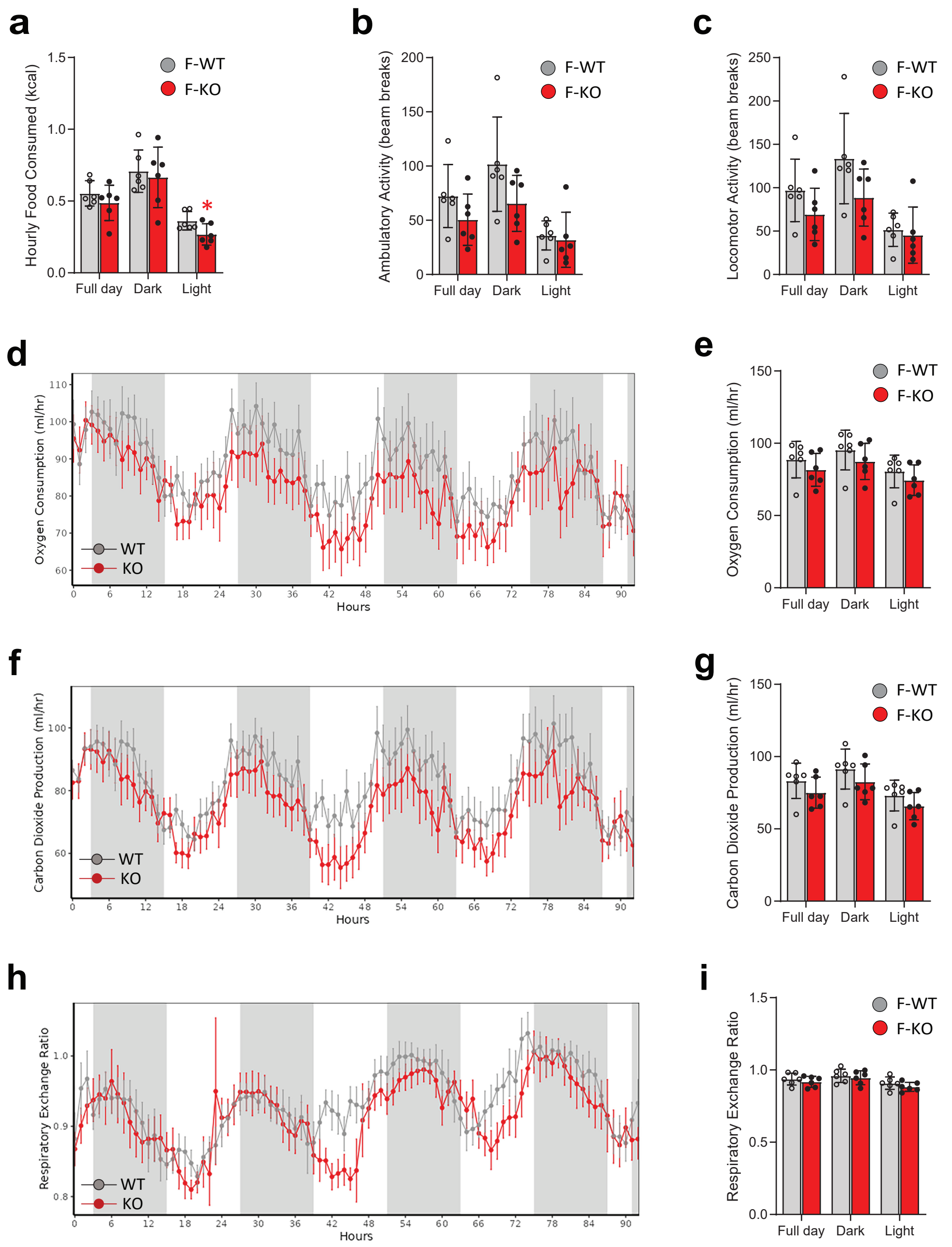

Fig. S7. Metabolic phenotypes in female KIAA1199 knockout mice and wild-type controls under normal diet (ND). Age-matched mice under chow diet were individually placed in metabolic cages (PhenoMaster) and allowed to acclimatize for 3 days before readings were taken. Different metabolic parameters were collected in a 4 days period and analyzed by Calr-Metabolism online program. Measurements were recorded on a cycle of dark (8:00 p.m. to 8:00 a.m.) and light (8:00 a.m. to 8:00 p.m.). Hourly food intake **(a)**, total ambulatory activity (sum of horizontal and vertical counts) over dark and light cycles **(b)**, total locomotor activity **(c)**, Oxygen consumption rate **(d-e)**, Carbon Dioxide production **(f-g)**, Respiratory exchange ratio (V_{CO2}/V_{O2}) **(h-i)** were compared between female WT (F-WT, n = 7) and female KO (F-KO, n = 7) mice. Data are expressed as means ± SD, the comparison between WT and KO groups are performed by two-tailed unpaired Student's t-test.

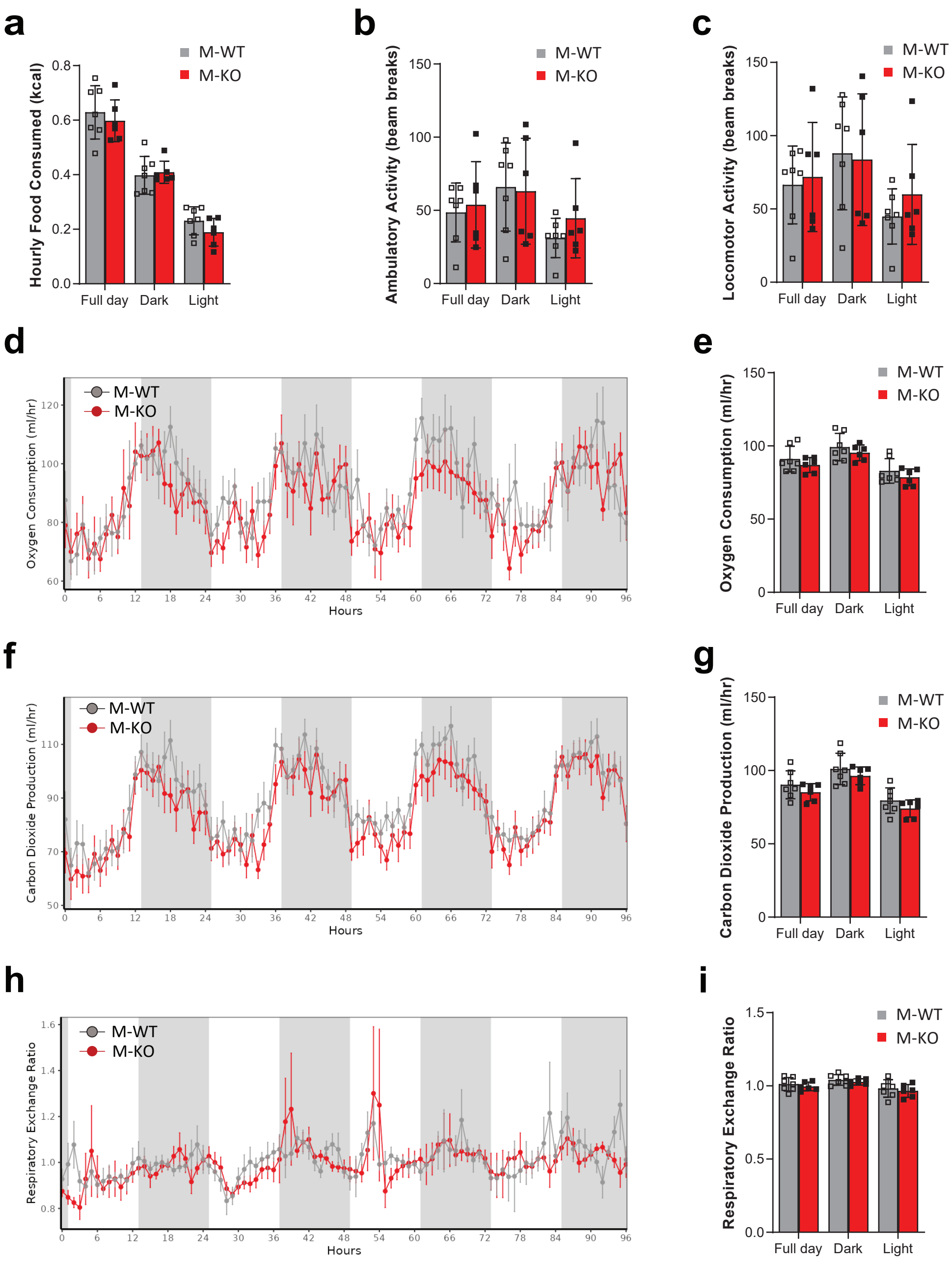

Fig. S8. Metabolic phenotypes in male KIAA1199 knockout mice and wild-type controls under normal diet (ND). Age-matched mice under chow diet were individually placed in metabolic cages (PhenoMaster) and allowed to acclimatize for 3 days before readings were taken. Different metabolic parameters were collected in a 4 days period and analyzed by Calr-Metabolism online program. Measurements were recorded on a cycle of dark (8:00 p.m. to 8:00 a.m.) and light (8:00 a.m. to 8:00 p.m.). Hourly food intake **(a)**, total ambulatory activity (sum of horizontal and vertical counts) over dark and light cycles **(b)**, total locomotor activity **(c)**, Oxygen consumption rate **(d-e)**, Carbon Dioxide production **(f-g)**, Respiratory exchange ratio (V_{CO2}/V_{O2}) **(h-i)** were compared between male WT (M-WT, n = 7) and male KO (M-KO, n = 7) mice. Data are expressed as means ± SD, the comparison between WT and KO groups are performed by two-tailed unpaired Student's t-test.

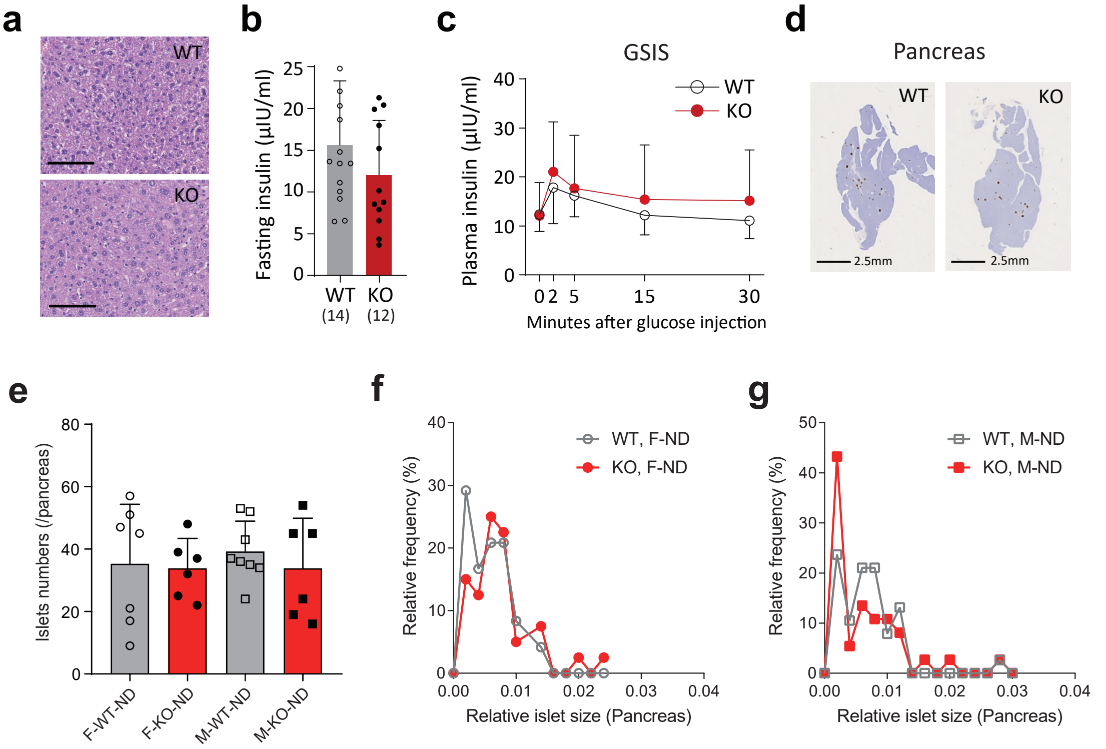

Fig. S9. KIAA1199 KO did not change the plasma insulin levels or insulin secretion by pancreas under normal diet. (a) Representative photomicrographs of histological sections of liver stained by H&E, scale bar =100 µm, n = 7. Fasting plasma levels of insulin **(b)** and glucose stimulated-insulin secretion (GSIS) **(c)** were checked in female KIAA11199 KO and WT female mice, n = 7. **(d)** Immunohistochemical staining for insulin to identify insulin-producing beta-cell (stained brown) in pancreas from age-matched female (F) and male (M) mice under normal diet (ND). Scale bar = 2.5 mm. The numbers of islets were counted **(e)**, and the sizes were analyzed by ImageJ and the size frequency were analyzed in GraphPad **(f-g)**.

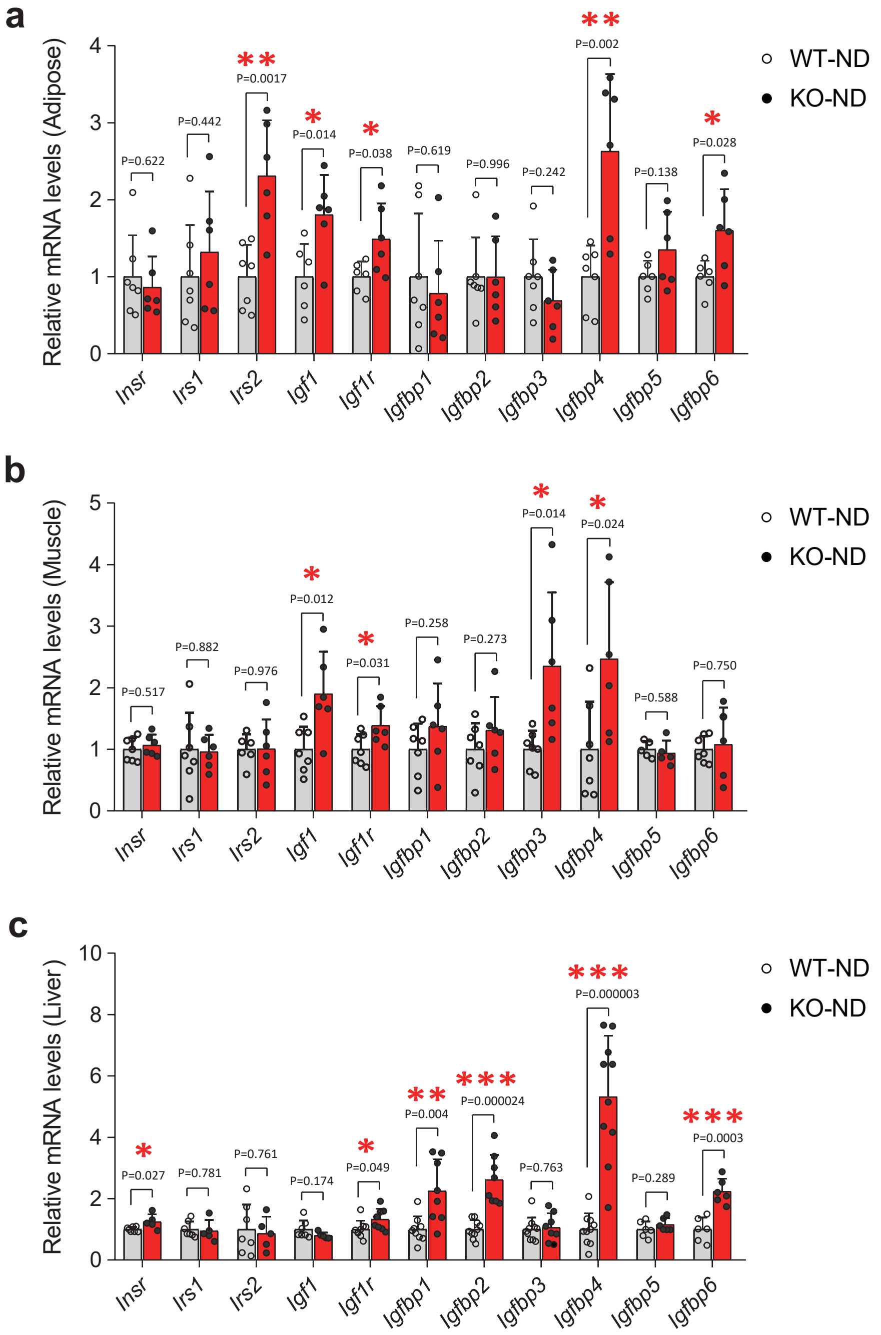

Fig. S10. Enlarged graph for Figure 3f with all the individual sample data. Steady-state expressions of genes associated with insulin signaling in adipose **(a)**, muscle **(b)** and liver **(c)** from female KIAA1199 KO and WT mice by real time qPCR. Data are expressed as means ± SD. Statistical difference between WT (n = 7) and KO (n = 6) for each gene is performed by two-tailed unpaired Student's t-test, *P < 0.05, **P < 0.01 and ***P < 0.001.

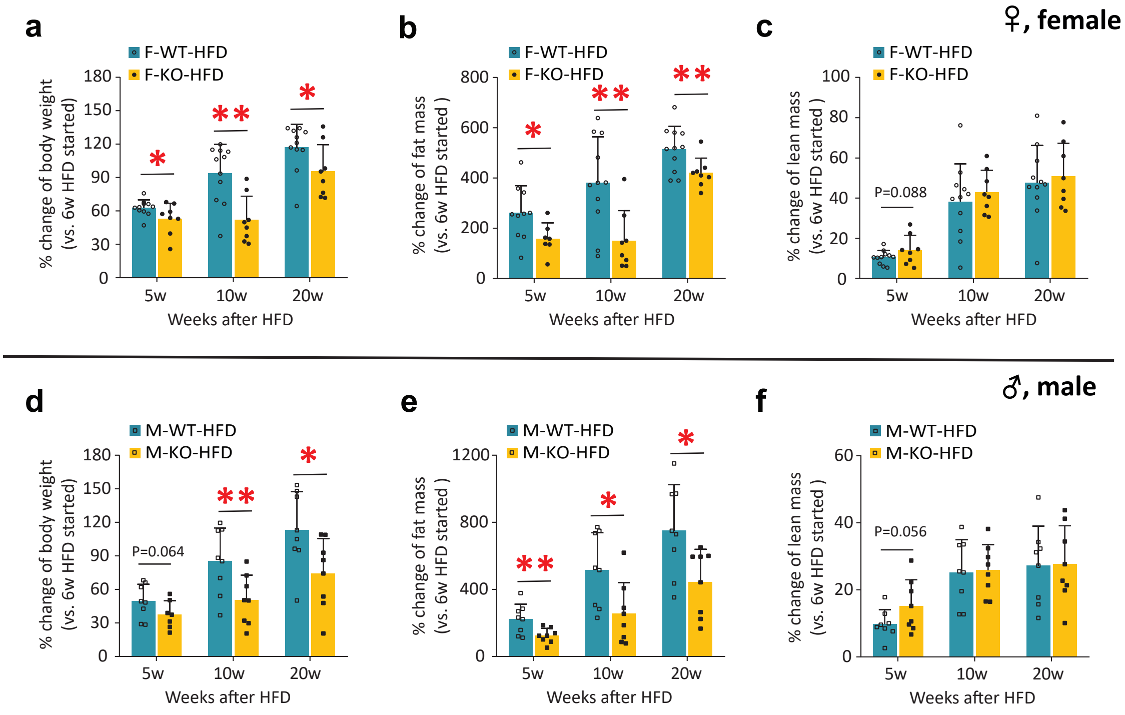

Fig. S11. KIAA1199 knockout (KO) mice significantly reduce body weight and fat formation. The percentage change of body weight **(a, d)**, fat mass **(b, e)** and lean mass **(c, f)** in female **(a-c)** and male **(d-f)** under HFD at age of 12, 18 and 24 weeks compare to the 6 weeks when HFD feeding started. n = 8 /group, Data are expressed as means ± SD. Statistical difference between WT and KO for each gene is performed by two-tailed unpaired Student's t-test, $*P < 0.05$, $**P < 0.01$.

Fig. S12

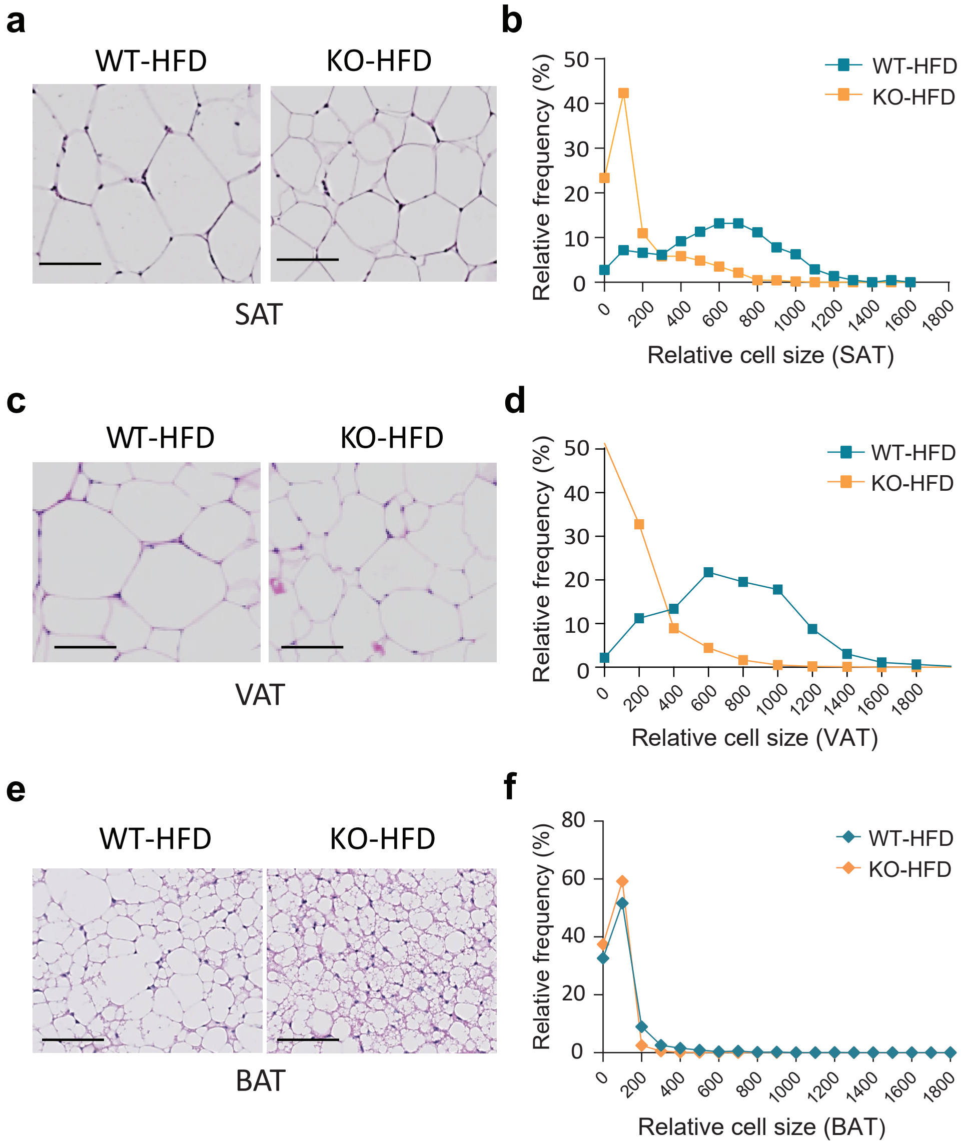

Fig. S12. KIAA1199 knockout reduces brown fat tissue adipose cell size. Photomicrographs of H&E staining histological sections of SAT **(a)**, VAT **(b)** and BAT **(c)** from KIAA1199 KO and WT under HFD and adipocyte size were measured by ImageJ (Adiposoft) and the size frequency were analyzed in GraphPad **(b, d, f)**. Scale bar = 100 µm, n = 7.

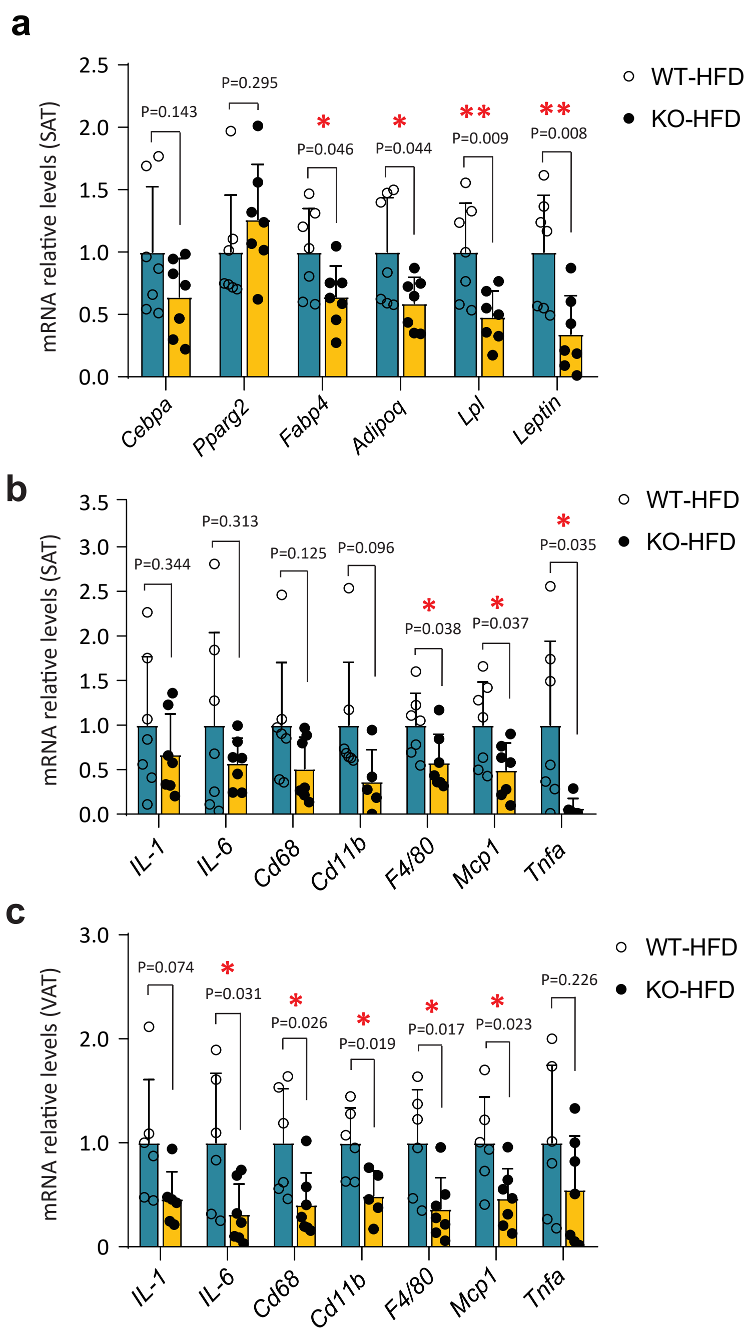

Fig. S13. Enlarged graph for Figure 4g, 4i & 4j with all the individual sample data. The adipocyte maker genes and inflammation-associated genes were analyzed in SAT **(a, b)** and VAT **(c)** in female KIAA1199 KO and WT mice under HFD by qPCR. Data are expressed as means ± SD. The comparison between WT (n = 7) and KO (n = 7) groups are performed by two-tailed unpaired Student's t-test. *P < 0.05, **P < 0.01.

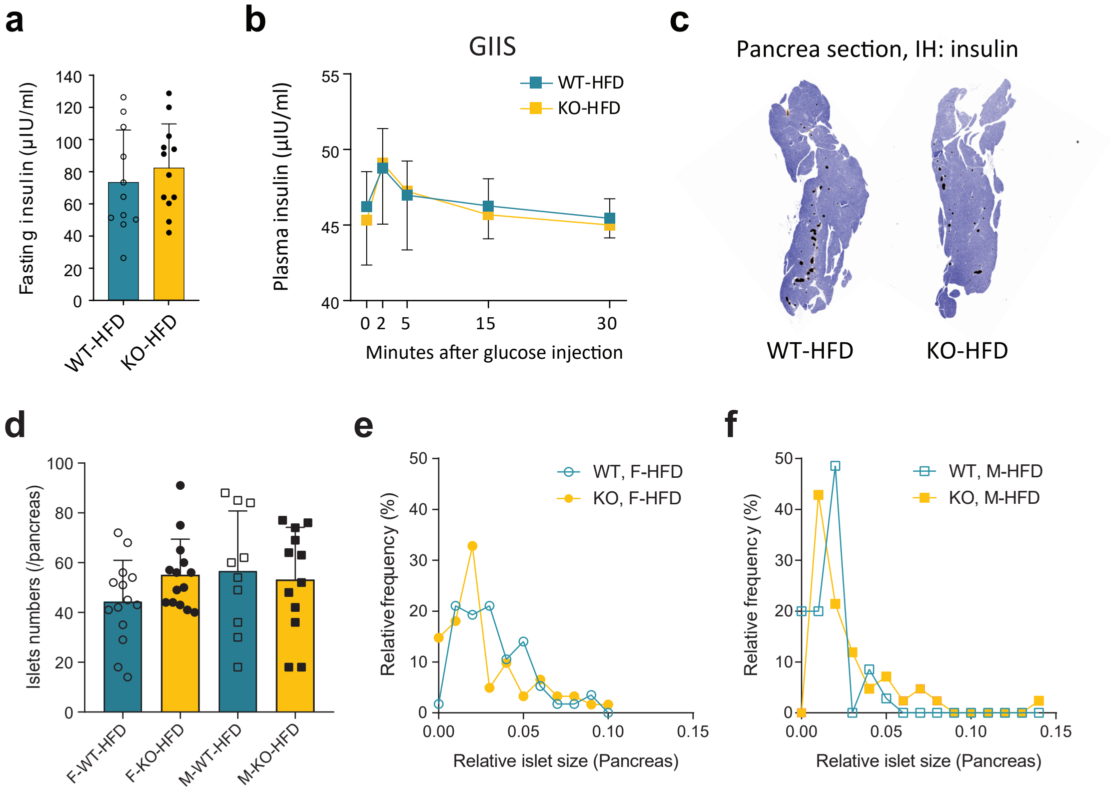

Fig. S14. KIAA1199 KO did not change the plasma insulin levels or insulin secretion by pancreas under high-fat diet (HFD). (a) Fasting plasma levels of insulin and glucose stimulated-insulin secretion (GSIS) **(b)** were checked in female KIAA11199 KO and WT female mice under HFD, n = 7. **(c)** Immunohistochemical staining for insulin to identify insulin-producing beta-cell (stained brown) in pancreas from age-matched female (F) and male (M) mice under HFD. The numbers of islets were counted **(d)**, and the sizes were analyzed by ImageJ and the size frequency were analyzed in GraphPad **(e-f)**.

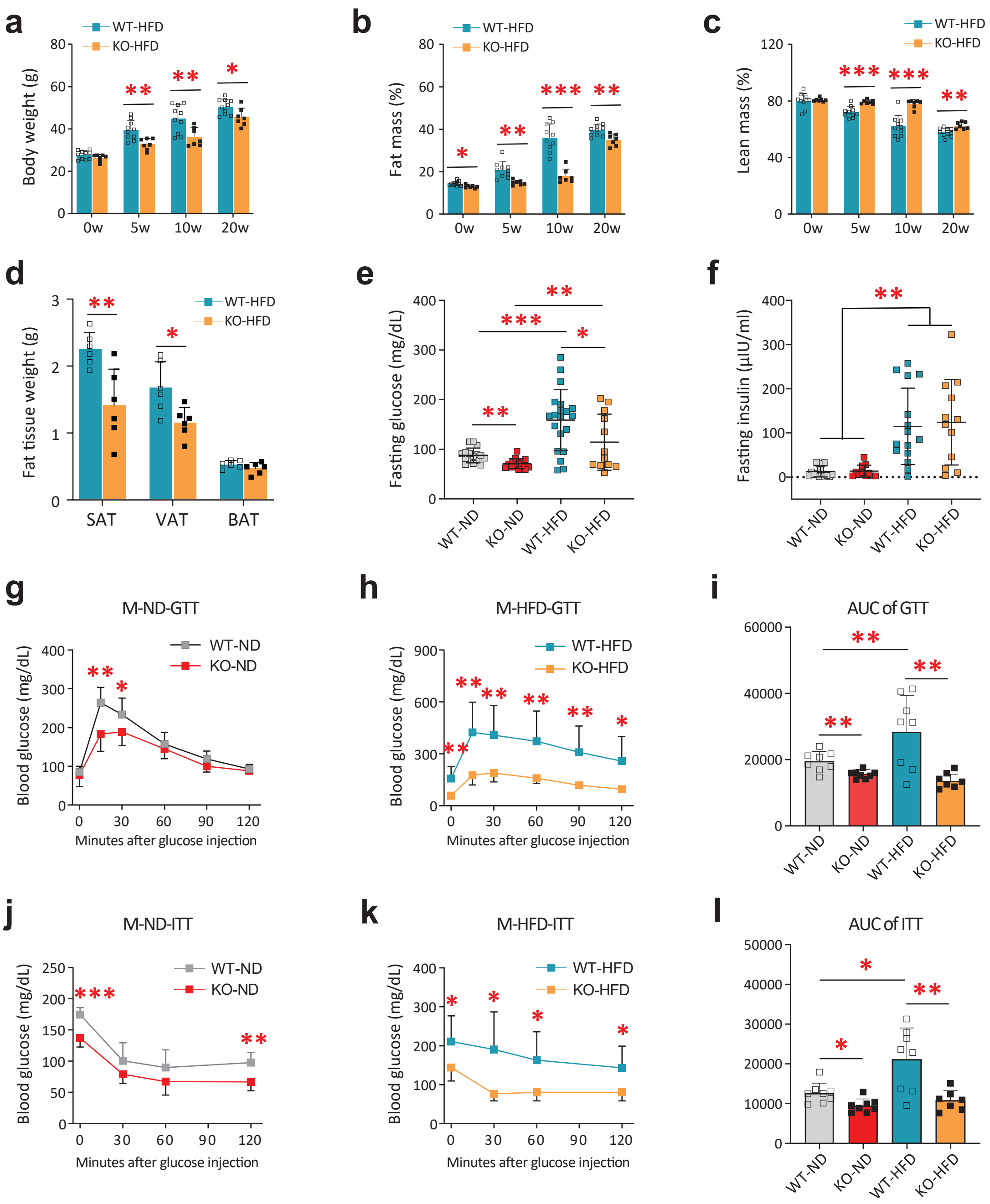

Fig. S15. KIAA1199 KO male mice have less weight and fat under high fat diet condition, and improve glucose and insulin metabolism. Age matched WT and KIAA1199 KO male mice were fed by high fat diet for 20 weeks. Total body weight (gram) **(a)**, body fat percentage **(b)**, body lean tissue percentage **(c)**, VAT: visceral adipocyte tissue, BAT: brown adipocyte tissue) from the corresponding sites were collected and weighted after 20 weeks high-fat diet (HFD) **(d)**. Fasting plasma insulin **(e)** and fasting plasma glucose **(f)** were measured in age matched WT and KIAA KO mice under normal diet (ND) and HFD. **(g-l)** Glucose tolerance test (GTT) and insulin tolerance test (ITT) were measured in matched male WT and KO mice under ND and HFD, the areas under curve (AUC) were calculated for each group. Data are expressed as means ± SD, Statistical difference was determined by one-way ANOVA with Dunnett's multiple tests to compare between groups, n = 6-15 / group, $*P < 0.05$, $**P < 0.01$, $***P < 0.001$.

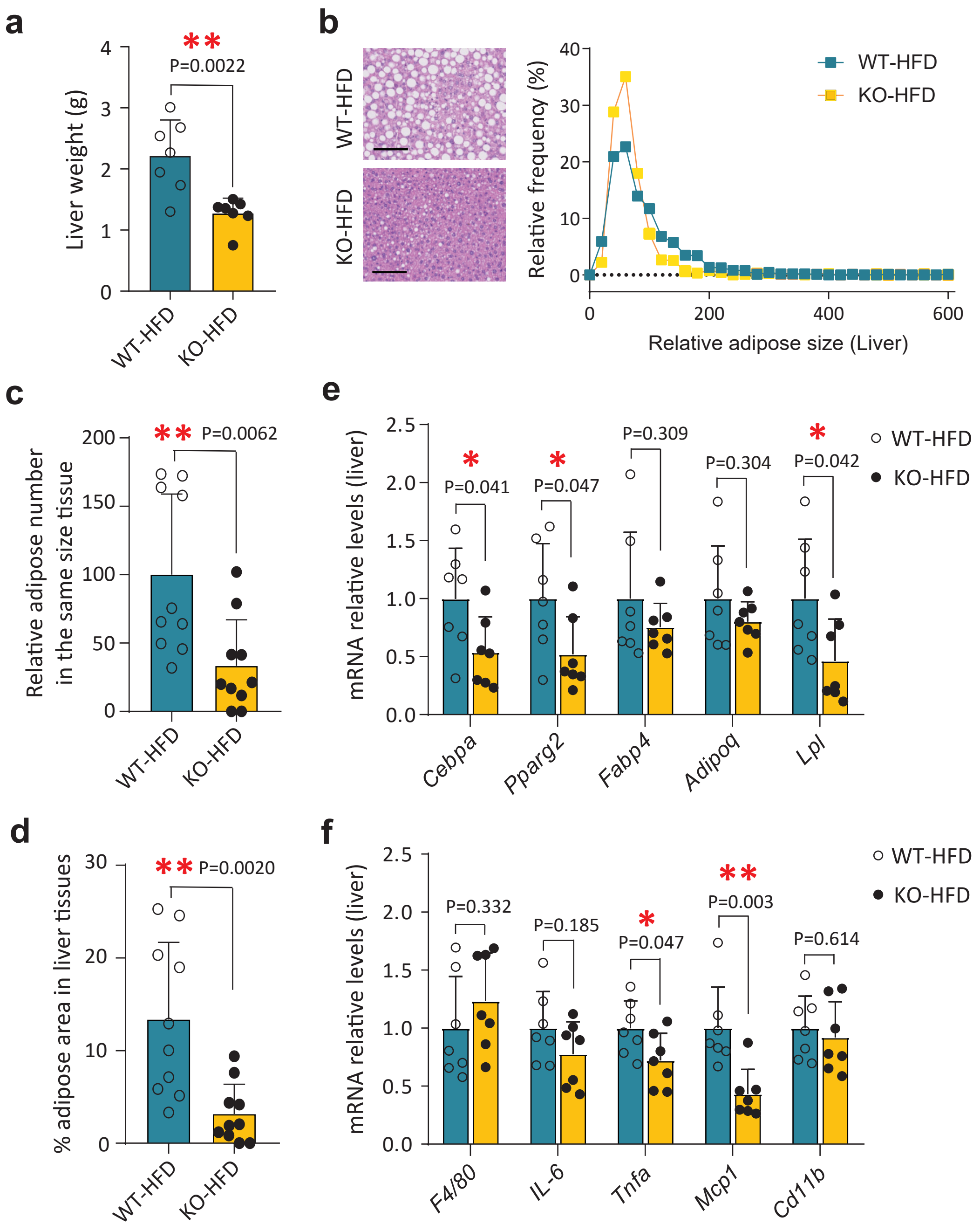

Fig. S16. KIAA1199 knockout mice are protected from HFD-induced hepatic steatosis. KIAA1199 knockout mice reduced liver weight **(a)**, liver adipose cell size **(b)**, liver adipose cell numbers **(c)** and percentage of adipose area in liver tissue **(d)** by immunohistochemistry analysis of liver tissue from wild-type and knockout mice after 24 weeks HFD feeding. **(e-f)** Enlarged graph for Figure 6N&O with all the individual sample data. Expression of adipocyte marker genes **(e)** and inflammation-associated marker genes **(f)** in the liver tissues measured in female KIAA1199 KO and WT mice under HFD by qPCR. Data are expressed as means ± SD. The comparison between WT (n = 7-10) and KO (n = 7-10) groups are performed by two-tailed unpaired Student's t-test. *P < 0.05, **P < 0.01.

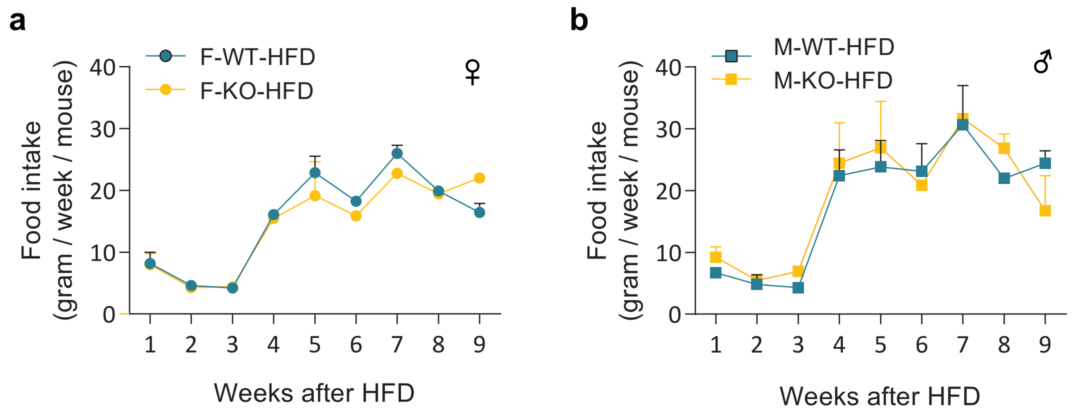

Fig. S17. KIAA1199 knockout (KO) mice did not change the food intakes under high-fat diet (HFD). Food intakes were traced in female **(a)** and male **(b)** at age of 6-14 weeks under HFD. Data are expressed as means ± SD, n = 8 /group.

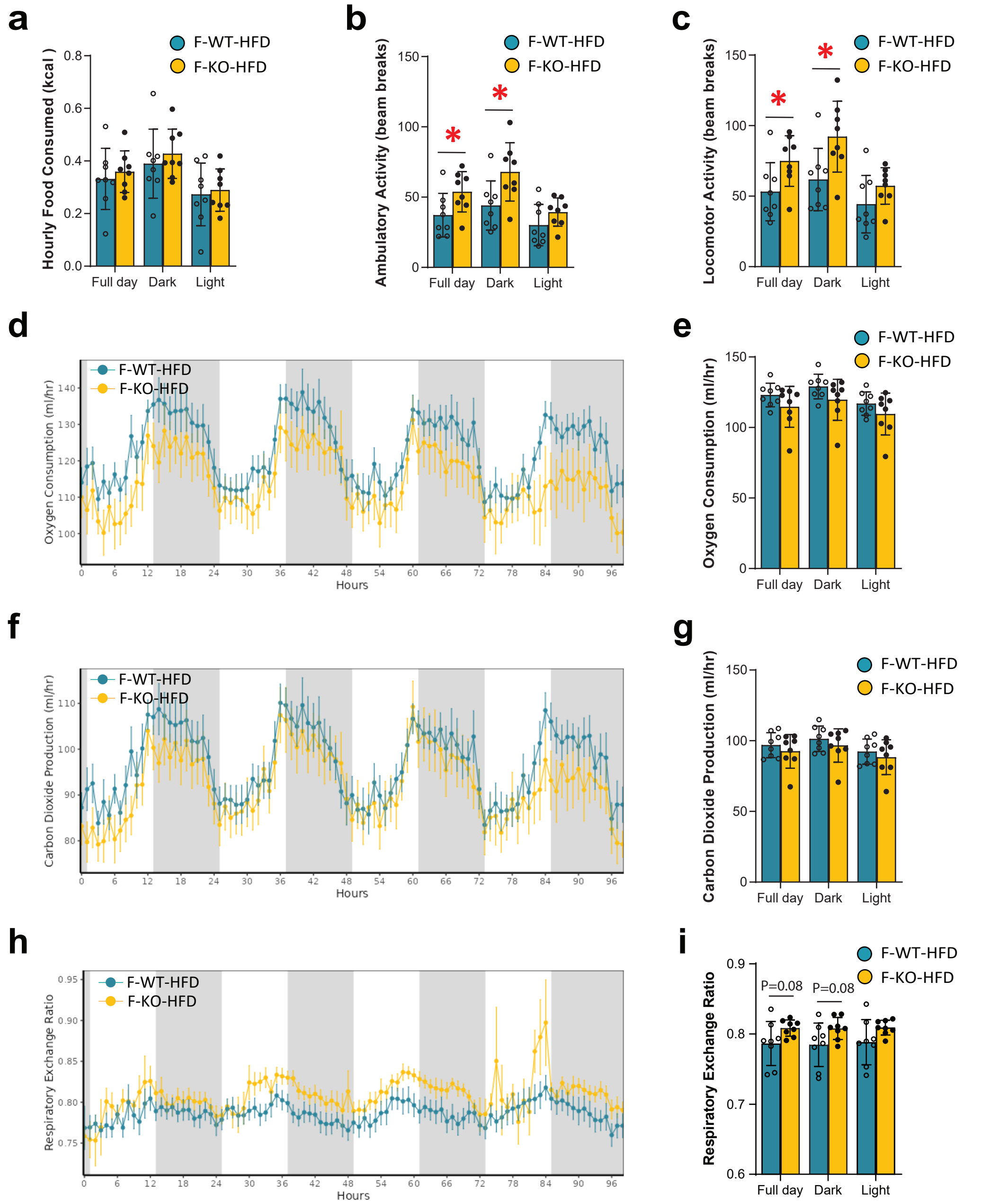

Fig. S18. The metabolic phenotypes of female KIAA1199 KO mice and WT controls under high fat diet (HFD). Age-matched mice under HFD were individually placed in metabolic cages (PhenoMaster) and allowed to acclimatize for 3 days before readings were taken. Different metabolic parameters were collected in a 4 days period and analyzed by Calr-Metabolism online program. Measurements were recorded on a cycle of dark (8:00 p.m. to 8:00 a.m.) and light (8:00 a.m. to 8:00 p.m.). Hourly food intake **(a)**, total ambulatory activity (sum of horizontal and vertical counts) over dark and light cycles **(b)**, total locomotor activity **(c)**, Oxygen consumption rate **(d-e)**, Carbon Dioxide production **(f-g)**, Respiratory exchange ratio (V_{CO2}/V_{O2}) **(h-i)** were compared between female WT (F-WT-HFD, n = 8) and female KO (F-KO-HFD, n = 8) mice. Data are expressed as means ± SD, the comparison between WT and KO groups are performed by two-tailed unpaired Student's t-test, *P < 0.05.

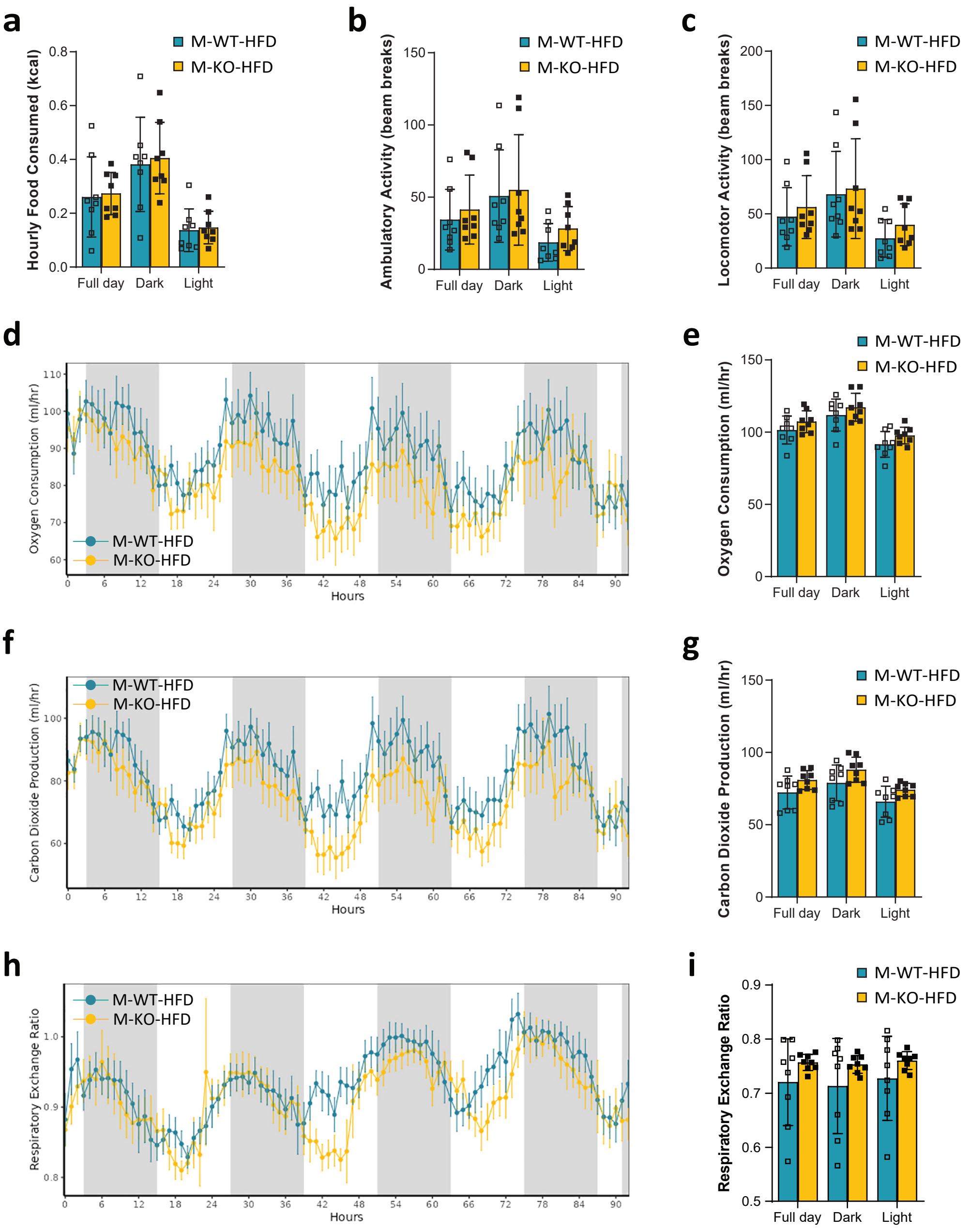

Fig. S19. The metabolic phenotypes of male KIAA1199 KO mice and WT controls under high fat diet (HFD). Age-matched mice under HFD were individually placed in metabolic cages (PhenoMaster) and allowed to acclimatize for 3 days before readings were taken. Different metabolic parameters were collected in a 4 days period and analyzed by Calr-Metabolism online program. Measurements were recorded on a cycle of dark (8:00 p.m. to 8:00 a.m.) and light (8:00 a.m. to 8:00 p.m.). Hourly food intake **(a)**, total ambulatory activity (sum of horizontal and vertical counts) over dark and light cycles **(b)**, total locomotor activity **(c)**, Oxygen consumption rate **(d-e)**, Carbon Dioxide production **(f-g)**, Respiratory exchange ratio (V_{CO2}/V_{O2}) **(h-i)** were compared between male WT (M-WT-HFD, n = 8) and male KO (M-KO-HFD, n = 8) mice. Data are expressed as means ± SD, the comparison between WT and KO groups are performed by two-tailed unpaired Student's t-test.

Fig. S20

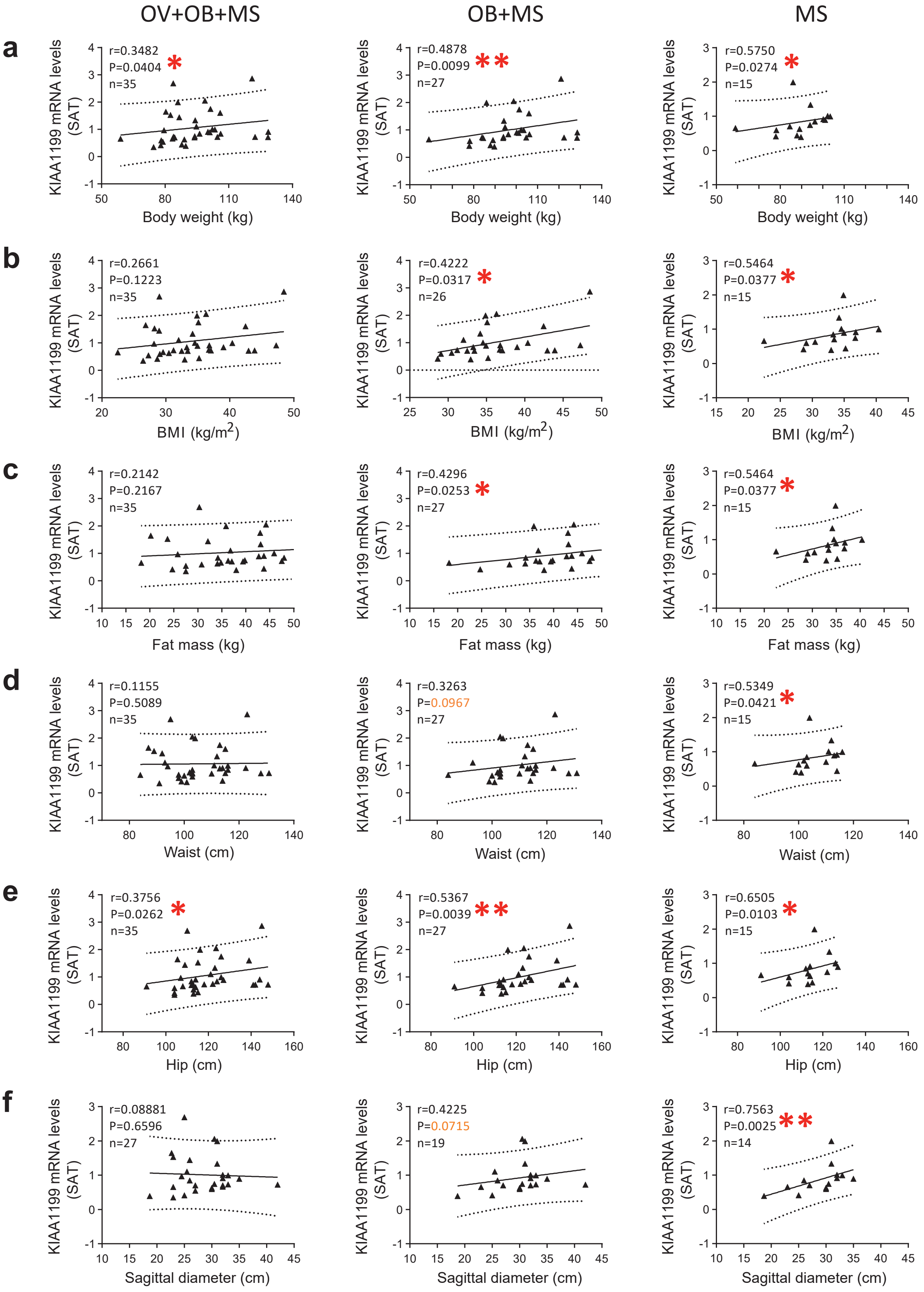

OV: Overweight; OB: Obesity; MS: Metabolic Syndrome

Fig. S20. The mRNA expression levels of KIAA1199 in subcutaneous adipose tissue (SAT)

correlate with body weight and obesity in different human groups. The mRNA expression levels of KIAA1199 in SAT were measured by real time qPCR in a cohort of 56 women that were grouped by body mass index (BMI) and metabolic syndrome as lean (LE), overweight (OV), obese (OB) or with metabolic syndrome (MS). The correlations of KIAA1199 mRNA levels in SAT with body weight **(a)**, BMI **(b)**, fat mass **(c)**, Waist circumference **(d)**, hip circumference **(e)** and sagittal diameter (SD) **(f)** were analyzed in OV+OB+MS, OB+MS and MS groups. Analysis of Spearman two-tailed correlation tests (r=correlation coefficient) were performed using GraphPad Prism software. The number of independent donors (n) in each correlation analysis is described in the results section and in each figure. *$P < 0.05$, **$P < 0.01$.

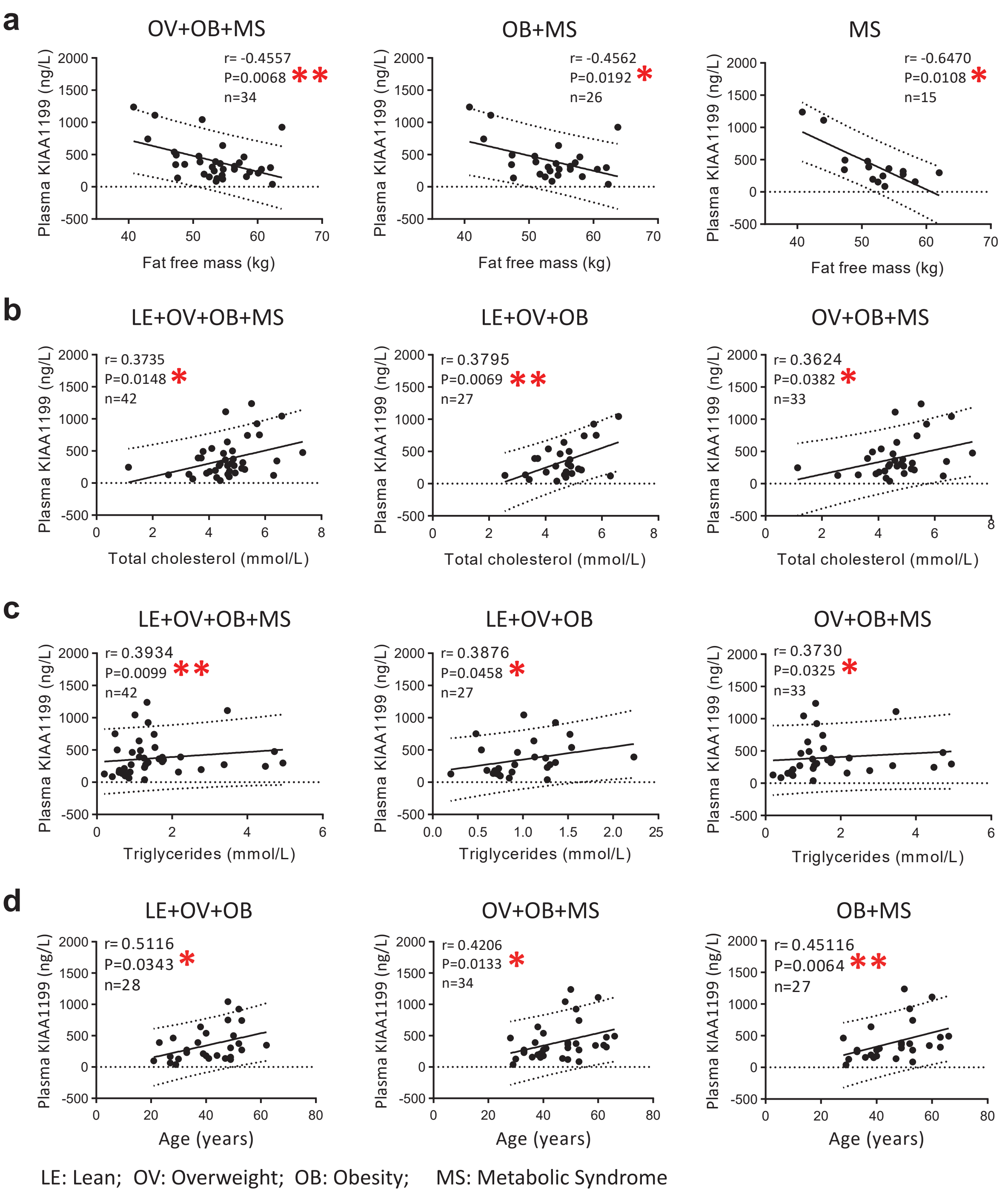

Fig. S21. The plasma concentrations of KIAA1199 correlate with fat free mass, total cholesterol and triglycerides concentrations in different human groups. The plasma concentrations of KIAA1199 were measured in a cohort of 56 women that grouped by body mass index (BMI) and metabolic syndrome as lean (LE), overweight (OV), obese (OB) or with metabolic syndrome (MS). The plasma concentrations of KIAA1199 correlates with fat free mass **(a)**, total cholesterol concentration **(b)**, triglycerides concentration **(c)** and age **(d)** in LE+OV+OB+MS, LE+OV+OB, OV+OB+MS, OB+MS or MS patients' groups. Analysis of Spearman two-tailed correlation tests (r=correlation coefficient) were performed using GraphPad Prism software. The number of independent donors (n) in each correlation analysis is described in the results section and in each figure. *P < 0.05, **P < 0.01.

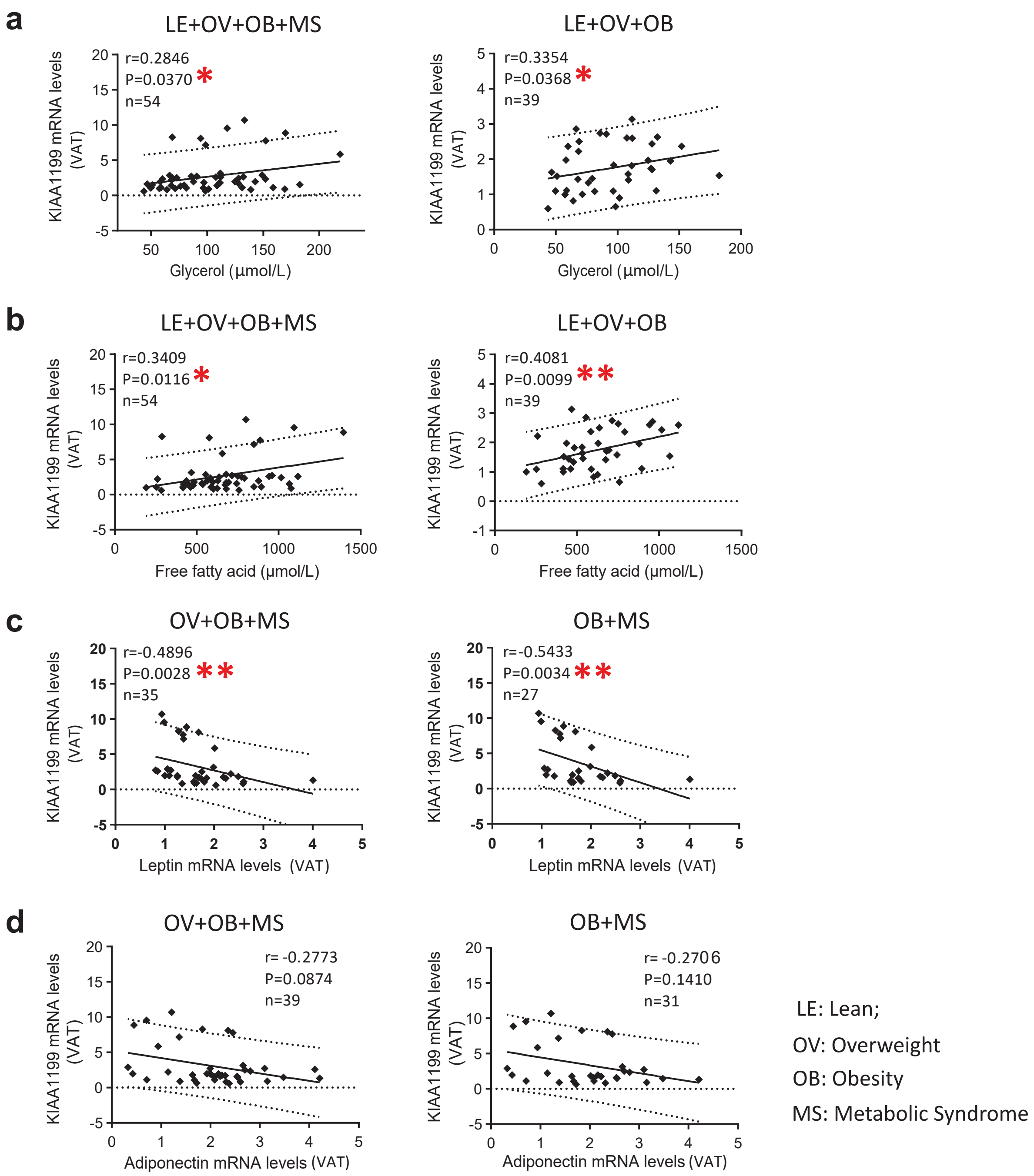

Fig. S22. The mRNA expression levels of KIAA1199 in visceral adipose tissue (VAT) correlate with free fatty acid concentration, glycerol concentration and leptin mRNA levels in different human groups. The expressions of KIAA1199 in VAT were measured in a cohort of 56 women that were grouped by body mass index (BMI) and metabolic syndrome as lean (LE), overweight (OV), obese (OB) or with metabolic syndrome (MS). The correlations of KIAA1199 mRNA in VAT with plasma free fatty acid **(a)**, plasma glycerol **(b)**, leptin mRNA levels in VAT **(c)** and Adiponection mRNA levels in VAT **(d)** were analyzed in LE+OV+OB, OV+OB+MS and OB+MS groups. Analysis of Spearman two-tailed correlation tests (r=correlation coefficient) were performed using GraphPad software. The number of independent donors (n) in each correlation analysis is described in the results section and in each figure. *P < 0.05, **P < 0.01.

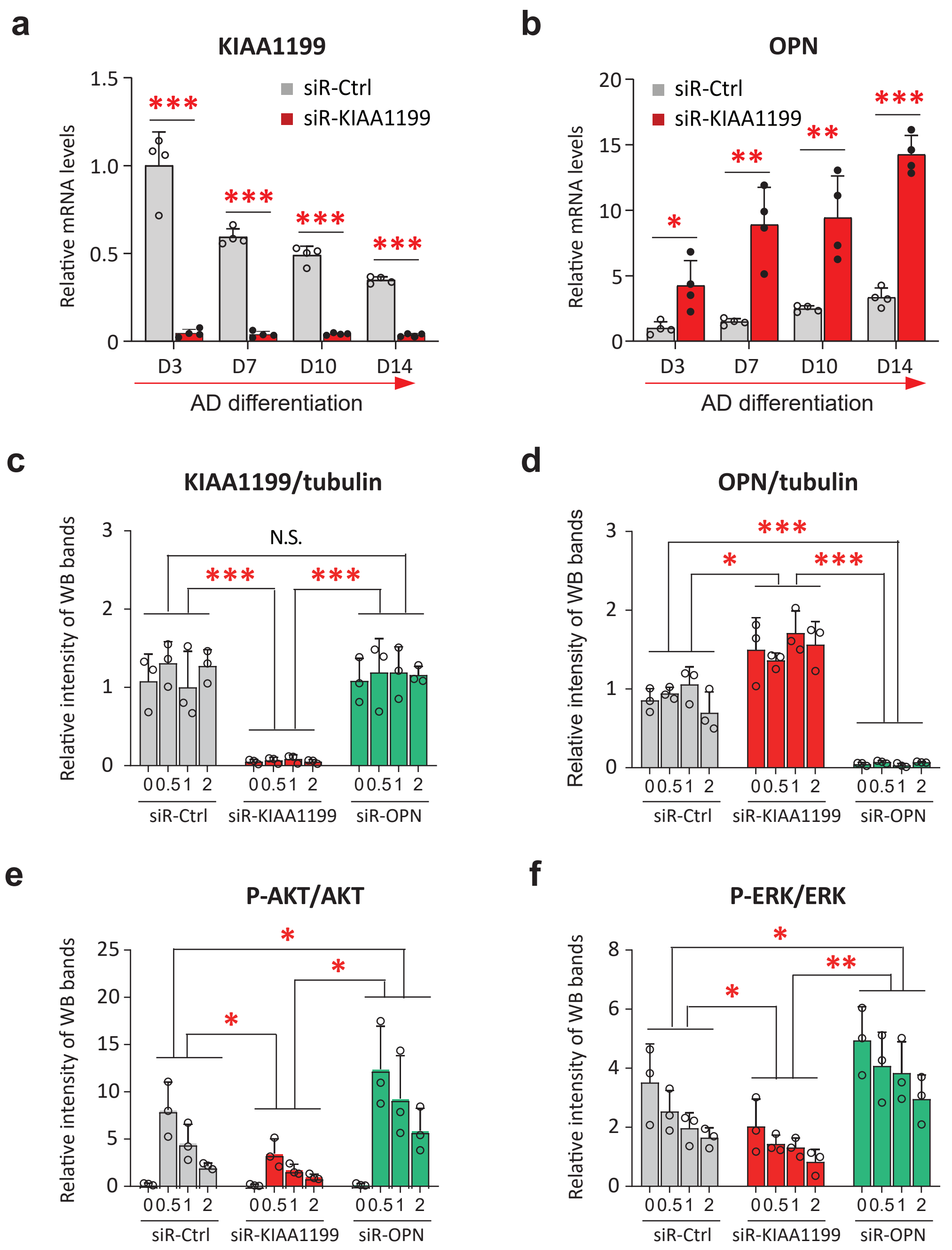

Fig. S23. Knockdown of KIAA1199 increases the expression of osteopontin (OPN), and regulates AKT and ERK pathways under adipocyte (AD) differentiation induction. Human bone mesenchymal stem cells (hBMSC) were cultured and transfected by specific siRNAs of hKIAA1199, then induced to adipocyte for 14 days. The expression of KIAA1199 **(a)** and OPN **(b)** were traced by qRT-PCR during AD differentiation. **(c-f)** Quantitation of Western blots bands intensity for KIAA1199 **(c)**, OPN **(d)**, P-AKT/AKT **(e)** and P-ERK/ERK **(f)** in Fig. 7g. Experiments were repeated at least three independent cohorts. Data are expressed as means ± SD. *$P < 0.05$, **$P < 0.01$, ***$P < 0.001$.

Supplementary Table 1. PCR primers for different genes used in the Real time PCR in the study

Species	Gene ID	Gene name	Forward primer (5’ → 3’)	Reverse primer (5’ → 3’)	Length (bp)	Gene Sequence ID
Human	*KIAA1199*	Cell migration-inducing and hyaluronan-binding [illegible]	TCTTTGGGCCACTGCTTCTTCACG	GTCTTGCCTGGGCTTGGGGATGTA	153	NM_001293298.1
Human	*OPN*	Osteopontin	CCAAGTAAGTCCAACGAAAG	GGTGATGTCCTCGTCTGTA	329	NM_000582
Human	*PPARG2*	Peroxisome proliferator-activated receptor gamma	TTCTCCTATTGACCCAGAAAGC	CTCCACTTTGATTGCACTTTGG	285	NM 015869.4
Human	*FABP4*	Adipocyte protein 2	GCCAGGAATTTGACGAAGTC	TGGTTGATTTTCCATCCCAT	84	NM_001442.2
Human	*ADIPOQ*	Adiponectin	GGGCCCCAGGCCGTGATGGCA	TCGGGGACCTTCAGCCCCGGGTA	102	NM_001177800
Human	*LPL*	Lipoprotein lipase	CTTGGAGATGTGGACCAGC	GTGCCATACAGAGAAATCTC	318	NM_000237.2
Human	*GAPDH*	Glyceraldehyde-3-phosphate dehydrogenase	GGCGATGCTGGCGCTGAGTAC	TGGTTCACACCCATGACGA	130	NM_002046
Human	*B2M*	Beta 2-microglobulin	CCTTGAGGCTATCCAGCGT	CCTGCTCAGATACATCAAACATG	510	NM_004048.2
Human	*ACTB*	Beta-actin	ATTGGCAATGAGCGGTTCCG	AGGGCAGTGATCTCCTTCTG	192	NM_001614.3
Mouse	*KIAA1199*	Cell migration-inducing and hyaluronan-binding [illegible]	GAGAAAAGACAATGGGCATA	TTCCCATCAGACCCAACA	338	NM_030728
Mouse	*Pparg2*	Peroxisome proliferator-activated receptor gamma	CTGATGCACTGCCTATGAGC	GGGTCAGCTCTTGTGAATGG	191	NM_011146.3
Mouse	*Fabp4*	Adipocyte protein 2	AGTGAAAACTTCGATGATTACATGAA	GCCTGCCACTTTCCTTGTG	101	NM_024406.2
Mouse	*Adipoq*	Adiponectin	GACGTTACTACAACTGAAGAGC	CATTCTTTTCCTGATACTGGTC	235	NM_009605
Mouse	*Lpl*	Lipoprotein lipase	CCAATGGAGGCACTTTCCA	CCACGTCTCCGAGTCCTCTC	61	NM_008509.2
Mouse	*Cebpa*	CCAAT/enhancer-binding protein alpha	AAGCCAAGAAGTCGGTGGA	CAGTTCACGGCTCAGCTGTTC	188	NM_001287514.1
Mouse	*Lep*	Leptin	GAGACCCCTGTGTCGGTTC	CTGCGTGTGTGAAATGTCATTG	139	NM_008493.3
Mouse	*IL-1b*	Interleukine 1 beta	GCAACTGTTCCTGAACTCAACT	ATCTTTTGGGGTCCGTCAACT	89	NM_008361.4
Mouse	*IL-6*	Interleukine 6	CTGCAAGAGACTTCCATCCAG	AGTGGTATAGACAGGTCTGTTGG	131	NM_001314054.1
Mouse	*CD68*	CD68 antigen	GGGGCTCTTGGGAACTACAC	GTACCGTCACAACCTCCCTG	167	NM_001291058.1
Mouse	*CD11b*	Integrin alpha-M/CD11 antigen-like family member B	ATGGACGCTGATGGCAATACC	TCCCCATTCACGTCTCCCA	203	NM_008401.2
Mouse	*F4/80/Adgre1*	Cell surface glycoprotein F4/80 / Adhesion G protein-coupled receptor E1	TCACTGTCTGCTCAACCGTC	TGCCATCAACTCATGATACCCT	254	NM_001355723.1
Mouse	*Mcp1/ccl2*	Monocyte chemotactic protein 1/Chemokine (C-C motif) ligand 2	GCTGGAGAGCTACAAGAGGATCACC	TCCTTCTTGGGGTCAGCACAGAC	102	NM_011333.3
Mouse	*Tnfa*	Tumor necrosis factor alpha	CCCTCACACTCAGATCATCTTCT	GCTACGACGTGGGCTACAG	61	NM_013693.3
Mouse	*Insr*	Insulin receptor	AGATGTCCCATCAAATATTGCCA	CCGGTGCACAAACTTCTTGG	631	NM_010568.3
Mouse	*Irs1*	Insulin receptor substrate 1	TCTACACCCGAGACGAACACT	TGGGCCTTTGCCCGATTATG	81	NM_010570.4
Mouse	*Irs2*	Insulin receptor substrate 2	CTGCGTCCTCTCCCAAAGTG	GGGGTCATGGGCATGTAGC	124	NM_001081212.2
Mouse	*Igf1*	Insulin-like growth factor 1	TCTGCTTGCTAAATCTCACTGT	ATAGGGACGGGGACTTCTGA	595	NM_010512.5
Mouse	*Igf1r*	insulin-like growth factor I receptor	GTGGGGGCTCGTGTTTCTC	GATCACCGTGCAGTTTTCCA	127	NM_010513.3
Mouse	*Igfbp1*	Insulin-like growth factor binding protein 1	CCCCAACTGCCACCAGTATT	TAAGCAGCATCACTCTGCCC	342	NM_008341.4
Mouse	*Igfbp2*	Insulin-like growth factor binding protein 2	CGCTACGCTGCTATCCCAAC	CATCACTGTCTGCAACCTGCT	120	NM_008342.4
Mouse	*Igfbp3*	Insulin-like growth factor binding protein 3	AGATGCGAGCTTAGAGCGG	GCGCGCACTGGGACA	290	NM_008343.2
Mouse	*Igfbp4*	Insulin-like growth factor binding protein 4	GTGGTGAACATTGAACGCCC	CACCCCATTCCTTTCCCACA	231	NM_010517.4

Mouse	*Igfbp5*	Insulin-like growth factor binding protein 5	ACGGCGAGCAAACCAAGATA	CTCAGCCTTCAGCTCGGAAA	134	NM_010518.2
Mouse	*Igfbp6*	Insulin-like growth factor binding protein 6	ACAGGGGGCTGTCATTTGAG	TGTAAAGGCCCAAGGGTGAC	107	NM_008344.3
Mouse	*B2m*	Beta 2-microglobulin	GCTATCCAGAAAACCCCTCAA	CATGTCTCGATCCCAGTAGACGGT	276	NM_009735.3
Mouse	*Actb*	Beta-actin	GATATCGCTGCGCTGGTCGTC	ACGCAGCTCATTGTAGAAGGTGTGG	217	NM_007393.5
Mouse	*36B4/Rplp0*	Acidic ribosomal phosphoprotein P0	TCCAGGCTTTGGGCATCA	CTTTATCAGCTGCACATCACTCAGA	74	NM_007475.5
